# Involvement of Local Lamellipodia in Endothelial Barrier Function

**DOI:** 10.1371/journal.pone.0117970

**Published:** 2015-02-06

**Authors:** Jerome W. Breslin, Xun E. Zhang, Rebecca A. Worthylake, Flavia M. Souza-Smith

**Affiliations:** 1 Department of Molecular Pharmacology and Physiology, Morsani College of Medicine, University of South Florida, Tampa, Florida, United States of America; 2 Department of Pharmacology, School of Medicine, Louisiana State University Health Sciences Center, New Orleans, Louisiana, United States of America; 3 Department of Physiology, School of Medicine, Louisiana State University Health Sciences Center, New Orleans, Louisiana, United States of America; University of Illinois at Chicago, UNITED STATES

## Abstract

Recently we observed that endothelial cells cultured in tightly confluent monolayers display frequent local lamellipodia, and that thrombin, an agent that increases endothelial permeability, reduces lamellipodia protrusions. This led us to test the hypothesis that local lamellipodia contribute to endothelial barrier function. Movements of subcellular structures containing GFP-actin or VE-cadherin-GFP expressed in endothelial cells were recorded using time-lapse microscopy. Transendothelial electrical resistance (TER) served as an index of endothelial barrier function. Changes in both lamellipodia dynamics and TER were assessed during baseline and after cells were treated with either the barrier-disrupting agent thrombin, or the barrier-stabilizing agent sphingosine-1-phosphate (S1P). The myosin II inhibitor blebbistatin was used to selectively block lamellipodia formation, and was used to test their role in the barrier function of endothelial cell monolayers and isolated, perfused rat mesenteric venules. Myosin light chain (MLC) phosphorylation was assessed by immunofluorescence microscopy. Rac1 and RhoA activation were evaluated using G-LISA assays. The role of Rac1 was tested with the specific inhibitor NSC23766 or by expressing wild-type or dominant negative GFP-Rac1. The results show that thrombin rapidly decreased both TER and the lamellipodia protrusion frequency. S1P rapidly increased TER in association with increased protrusion frequency. Blebbistatin nearly abolished local lamellipodia protrusions while cortical actin fibers and stress fibers remained intact. Blebbistatin also significantly decreased TER of cultured endothelial cells and increased permeability of isolated rat mesenteric venules. Both thrombin and S1P increased MLC phosphorylation and activation of RhoA. However, thrombin and S1P had differential impacts on Rac1, correlating with the changes in TER and lamellipodia protrusion frequency. Overexpression of Rac1 elevated, while NSC23766 and dominant negative Rac1 reduced barrier function and lamellipodia activity. Combined, these data suggest that local lamellipodia, driven by myosin II and Rac1, are important for dynamic changes in endothelial barrier integrity.

## Introduction

The endothelium of capillary and postcapillary venules is a semi-permeable barrier critical for normal blood-tissue exchange of fluids and solutes. During inflammation, this barrier becomes compromised, allowing increased transport of plasma proteins into the surrounding tissues. When inflammation is prolonged and uncontrolled, microvascular hyperpermeability can cause edema and disrupt normal tissue homeostasis.

The junctional space between endothelial cells is thought to be the predominant pathway for transendothelial flux of macromolecules during inflammation [1]. In postcapillary venules, the adhesive strength of junctional proteins such as VE-cadherin is essential to maintain the barrier [2]. During barrier compromise due to inflammatory mediators, current theory suggests that centripetal tension can put stress on the junctions and limit their strength, delaying recovery of normal barrier integrity [3–5]. Various inflammatory stimuli promote development of actin stress fibers, which are thought to increase centripetal tension [6,7]. In contrast, agents that reduce permeability, such as the bioactive lipid sphingosine-1-phosphate (S1P), have been reported to increase the number of cortical actin fibers, stabilizing the cell periphery and strengthening junctions between endothelial cells [8,9].

Actin-mediated contraction in endothelial cells is promoted by phosphorylation of myosin regulatory light chains (MLC) on Thr-18/Ser-19, which is determined by the activities of MLC kinase (MLCK) and MLC phosphatase (MLCP). Inhibition of MLCK was reported to decrease baseline permeability in isolated coronary venules [10], and attenuate neutrophil-induced hyperpermeability [11]. Likewise, deletion of the long chain MLCK in mice attenuated microvascular leakage caused by severe burns [12]. While MLCK inhibition did not prevent thrombin-induced endothelial barrier dysfunction in cultured endothelial cells, it did significantly accelerate the recovery toward baseline [4].

Many of the same agents that produce endothelial hyperpermeability have also been reported to activate the small GTPase RhoA, leading to downstream activation of ROCK. In turn, ROCK phosphorylates the targeting and regulatory subunit of MLCP, MYPT-1 leading to MLCP inactivation, facilitating the accumulation of phosphorylated MLC [6,7,13–17]. Inhibition of MLCK or ROCK has been reported to decrease actin stress fiber formation, typically observed in fixed cells by labeling F-actin with a fluorochrome-bound phalloidin [3,9,11,13–20]. These and other studies reporting coincidence of actin stress fiber formation with elevated endothelial permeability, and that inhibition of either MLCK or ROCK attenuates both in tandem, provide the main support for the concept that actin stress fibers contribute to the weakening of the endothelial barrier.

Agents that reduce endothelial permeability, such as S1P and cAMP analogs, are reported to activate the small GTPase Rac1, which promotes cortical actin structures and stabilizes intercellular junctions [7,21–24]. Thrombin-induced endothelial hyperpermeability is associated with decreased Rac1 activity [25]. In addition, NSC23766, which blocks Rac1 activation by the guanine exchange factor Tiam1, increases endothelial permeability [26,27]. Conversely, stimulation of the Epac1-Rap1 pathway by cAMP promotes Tiam1-mediated Rac1 activation, stabilizing cortical actin and junctions between endothelial cells [19,24,25,28,29]. Combined, these reports support the concept that a stable cortical actin ring enhances the endothelial barrier.

To date, our knowledge of the actin cytoskeleton’s role in endothelial barrier function has come mainly from observations of fixed specimens, representing snapshots in time and providing little information on spatial dynamics. We aimed to better understand how the dynamics changes in the actin cytoskeleton in response to inflammatory mediators, and how actin stress fibers form in endothelial cells, which required precise visualization of the actin cytoskeleton in living cells. GFP-actin was previously shown to be a suitable probe for actin cytoskeletal dynamics in live cells [30,31]. We optimized a protocol to express GFP-actin in endothelial cells, enabling study of actin in live cells before and after treatment with inflammatory stimuli [32]. In our initial study, we observed the fluid nature of the endothelial actin cytoskeleton. One surprising finding was that confluent endothelial cells exhibited frequent protrusion and retraction of local lamellipodia, and that thrombin inhibited the formation of lamellipodia [32]. Based on these initial data, we hypothesized that local lamellipodia formation and withdrawal directly correlates with barrier integrity. We utilized thrombin, a very robust stimulator of endothelial barrier dysfunction in vitro, and S1P, a robust barrier-enhancing agent, to test our hypothesis. We assessed how these agents act upon the actin cytoskeleton and cell-cell junctions over time by transfecting endothelial cells with GFP-actin or VE-cadherin-GFP, respectively, and correlated these data with changes in barrier function. In addition, we found that the myosin II inhibitor blebbistatin could selectively inhibit local lamellipodia in endothelial cells without significantly changing cortical actin or stress fibers, and used this compound to directly test the role of local lamellipodia in endothelial barrier function. We also investigated the potential roles of MLC phosphorylation and Rho family GTPase activation.

## Materials and Methods

### Ethics Statement

All animal protocols were performed in strict accordance with the U.S. Animal Welfare Act, U.S. Public Health Service Policy on the Humane Care and Use of Laboratory Animals, and the *Guide for the Care and Use of Laboratory Animals*. All animal experiments in this study were performed after approval from the Louisiana State University Health Sciences Center—New Orleans Institutional Animal Care and Use Committee (Permit Number: 2968). All surgery was performed after the rats were anesthetized with ketamine/xylazine, and all efforts were made to minimize suffering.

### Materials

Clonetics Human umbilical vein endothelial cells (HUVEC), Endothelial Growth Medium-2MV (EGM2MV), Endothelial Basal Medium (EBM), and HUVEC Nucleofector transfection kits were obtained from Lonza (Basel, Switzerland). The pCMV-GFP-β-actin (herein the protein product is referred to as GFP-actin) plasmid vector was generously provided by Dr. A. Wayne Orr (Department of Pathology, Louisiana State University Health Sciences Center-Shreveport). The pVE-cadherin-GFP plasmid [33,34] was generously provided by Dr. Daniel Riveline (Institut de Science et d'Ingénierie Supramoléculaires, Université de Strasbourg, France). The pcDNA3-GFP-Rac1 (wild type) and pcDNA3-GFP-Rac1 T17N (dominant negative) plasmids were obtained from Cell Biolabs (San Diego, CA). Sphingosine-1-phosphate (S1P), (-)blebbistatin, (+)blebbistatin, NSC23766, and Mouse anti-GFP (clone 3F8.2) were purchased from Merck-Millipore (Billerica, MA). Alexa Fluor 488-donkey anti-rabbit IgG antibody (A21206), Alexa Fluor 647-donkey anti goat IgG antibody (A21447), Alexa Fluor 488-albumin, Alexa Fluor 594-phalloidin, Texas Red-phalloidin, and Hoechst 33342 were purchased from Invitrogen (Carlsbad, CA). Goat anti-VE-cadherin (sc-6458) and HRP-conjugated-Mouse anti-β-actin (sc-47778 HRP) was purchased from Santa Cruz Biotechnology, Inc. (Santa Cruz, CA). Rabbit anti phospho-MLC2-T18/S19 (#3674) was obtained from Cell Signaling Technology (Boston, MA). HRP-conjugated donkey anti-mouse IgG secondary antibodies were purchased from Jackson Immunoresearch (West Grove, PA). Thrombin and all other chemicals unless otherwise noted, were purchased from Sigma-Aldrich (St. Louis, MO).

### Cell Culture and Transfection

Passage 2–6 HUVEC were used for all experiments. HUVEC were routinely cultured in EGM2MV. HUVEC were transfected using the Nucleofector II system (Lonza). Briefly, cells grown to 80% confluence were trypsinized and pelleted, and 5 x 10^5^ cells were mixed with 0.2 μg of plasmid vector and 100 μl of Nucleofector solution in a Nucleofection cuvette, using program A-34 or A-23. Immediately after, 500 μl of EGM2MV was added and the cells were allowed to recover for 15 min at 37°C. The cells were then seeded onto gelatin-coated 35 x 22 mm glass #1 coverslips or MatTek 35 mm #1 glass bottom dishes (MatTek Corp., Ashland, MA) for live cell imaging studies, 8W1E ECIS arrays (Applied Biophysics, Troy, NY) for endothelial barrier function studies, or 100 mm culture dishes for Western blotting.

### Assessment of GFP-Actin and Native Actin Levels

Cell lysates were obtained from HUVEC expressing GFP-actin and untransfected HUVEC as previously described [3,13]. Lysates were mixed with NuPAGE sample buffer containing reducing agent, and the samples were run on pre-cast 10% Bis-Tris gels (Invitrogen, Carlsbad, CA). Proteins were electrically transferred to nitrocellulose membranes, which were probed with HRP-conjugated mouse anti-β-actin (1:200 dilution) or mouse anti-GFP (1:1000) followed by HRP-conjugated anti-mouse secondary antibodies (1:5000). Bands were visualized using WestPico Supersignal reagent (Pierce, Rockford, IL) and a Bio-Rad VersaDoc Model 5000 imaging system.

To determine whether GFP-actin was incorporated ubiquitously into actin fibers, we labeled F-actin in GFP-actin-expressing HUVEC with Alexafluor-594-phalloidin. Briefly, cells grown on coverslips were fixed in 4% paraformaldehyde and permeabilized with 0.1% Triton X-100. The cells were incubated with 165 nM Alexafluor-594-phalloidin for 40 minutes, washed 3 times with PBS, and mounted on glass slides with Vectashield containing DAPI (Vector Labs, Burlingame, CA) for immunofluorescence microscopy. Standard images (non-confocal) were acquired with an ASI RAMM system equipped for immunofluorescence imaging (Applied Scientific Instrumentation, Eugene, OR).

### Live Cell Imaging Protocols

Experiments were performed using either a Nikon Eclipse TE-2000U inverted microscope (Nikon Instruments, Melville, NY) or an ASI Rapid Automated Modular Microscope with a motorized stage and CRISP autofocus system (Applied Scientific Instrumentation, Eugene, OR). Each was equipped the following: a Sutter Instruments Lambda LS 300 W xenon lamp, Lambda 10–3 excitation filter wheel with SmartShutter (Sutter Instruments, Novato, CA) and S492, S572, and D350 filters, a dichroic 2002bs emitter (61002m; Chroma Technology Corporation, Bellows Falls, VT), 40x ELWD and 100X oil objectives (Nikon Instruments), and a Photometrics CoolSNAP HQ2 camera (Photometrics, Tucson, AZ). The Nikon system was connected to a Dell computer with Nikon Elements AR software for image acquisition, while the ASI system was equipped with a Mac Pro computer and Micromanager software [35]. For some of the experiments during the initial stages of this study, Metamorph 6.2 software (Molecular Devices, Sunnyvale, CA) was used for image acquisition on the Nikon system. The cell-covered coverslips or MatTek dish was attached to a Warner Instruments open diamond bath (RC22 or RC37, respectively) using vacuum grease to form a chamber, which was mounted into a PH-1 heated stage adapter. The input line on the chamber was connected serially to an inline solution heater (SH-27B) and a gravity reservoir containing albumin physiological salt solution (APSS: NaCl, 120 μM; KCl, 4.7 μM; CaCl2·2H2O, 2 μM; MgSO4·7H2O, 1.2 μM; NaH2PO4, 1.2 μM; Na pyruvate, 2 μM; glucose, 5 μM; EDTA, 0.02 μM; MOPS, 3 μM and purified BSA 1 g/100ml). The stage adapter and inline heater were maintained at 37°C by a Warner Instruments TC324B temperature controller (Warner Instruments, Hamden, CT). The flow rate of APSS over the cells was controlled by a pinch clamp and kept at ~0.5 mL/min. Fields of view for study were chosen when cells had sufficient fluorescence emission to visualize GFP-actin filaments or junctional areas containing VE-cadherin-GFP. Areas in which emission was very high were avoided as this decreased resolution of subcellular structures. After acquiring initial brightfield and fluorescent (S492 excitation) images, a time-lapse image set was collected with 1–2 s exposures every 15 s for up to 2.5 h. Images were typically acquired in a 1392 x 1040 format in 14 bit mode at 20 MHz with 2 x 2 binning and gain set at 1, although some were taken with no binning. On the Nikon system the focus knobs were locked for the experiments, however in some cases focus needed to be re-adjusted during the time course and the interval between images was slightly altered. This was taken into account in subsequent analyses. The CRISP autofocus on the ASI microscope system automatically corrected problems with focus. Pharmacological agents were applied to the reservoir and bath by a micropipette, and thus were not washed out in these experiments.

### Image Analysis

Time-lapse image stacks were saved in Nikon ND2 or TIF format for storage. The image stacks were exported as TIF files for analysis using Fiji/ImageJ software [36]. Brightness and contrast were adjusted for easier display but the original pixel intensity data were not altered. To assess actin dynamics at the cell periphery, we determined the frequency of lamellipodia formation (protrusion frequency). Filopodia were very infrequent with confluent endothelial cells, and typically formed as the result of the withdrawal of a local lamellipodium and thus were not quantified in this measure. The protrusion frequency was quantified by counting the number of local lamellipodia that formed on the perimeter of the entire cell during a particular time period. The protrusion frequency was normalized to the cell perimeter, which was estimated by drawing lines around the perimeter and measuring them using Fiji. Protrusion frequency is expressed as #/μm perimeter/time.

Kymograph analysis of local lamellipodia was used to evaluate their motile dynamics (S1 Fig.). A single-pixel width line was drawn perpendicular to the edge of a cell (S1A Fig.), and this region was extracted from each image of the time-lapse to generate a montage of the region over time (S1B Fig.). In this panel, the streaks that move rightward and upward represent actin-rich protrusions, while the continuous lines that tend to move rightward and downward represent actin fibers moving toward the center of the cell. To assess protrusion dynamics, a line was drawn on each upward/rightward streak (S1C Fig.). This was easiest when the adjacent cell did not express GFP-actin, but was also achievable when an adjacent cell also expressed GFP-actin by scrolling through time-lapse images to help identify events that were lamellipodia. Using the ImageJ Measure function, bounding rectangle data were acquired for each line, from which we determined the protrusion time and protrusion distance (S1D Fig.). The protrusion velocity was calculated as the protrusion distance/protrusion time. The withdrawal distance, withdrawal time, and withdrawal velocity were also measured for retracting lamellipodia in the same way with lines drawn along the cell edge. For all cells, 6–9 kymographs were generated to produce a representative sample of lamellipodia for study.

Dynamics of actin fibers were also assessed using kymograph analysis (S2 Fig.). For this analysis, a line was drawn across the entire width of a cell (S2A Fig.). In cells where very few fibers were present during baseline, we drew two lines spanning different parts of the cell to capture a representative sample. A kymograph was generated from each line in which the x-axis represented distance and the y-axis time (S2B Fig.). Lines formed by the presence of stress fibers were identified and annotated (S2C Fig.), and the ImageJ Measure function was used to obtain bounding rectangle data. The lateral velocity of the actin fibers was calculated as the distance/time, with movements toward the cell center assigned a positive value and movements toward the periphery a negative value. The number of actin fibers within the kymograph during each time point was also obtained from this data.

Movies files (AVI format) were generated from the time-lapse images with FIJI/ImageJ software, using JPEG compression. Brightness and contrast were adjusted to optimize view of the structures containing GFP-actin or VE-cadherin-GFP. When it was necessary to reduce file size, every other frame was removed from the time-lapse stack so that the interval between frames was increased to 0.5 s.

### Endothelial Cell Monolayer Barrier Function

Barrier function of HUVEC monolayers was determined using an Electrical Cell-Substrate Impedance Sensor (ECIS) Model 1600R (Applied Biophysics, Troy, NY), as previously described [27,37]. Briefly, 1.5 x 10^5^ cells were seeded in 400 μl of medium per well onto gelatin-coated gold-film surface electrode arrays (8W1E). The cells were allowed to attach overnight and form confluent monolayers. A 1-μA AC signal at 4000 Hz was applied from an approximate current source. Voltage was monitored across the cell-covered electrodes and its phase relative to the applied current, providing a report of total impedance. Treating the cell-electrode system as a series RC circuit, the ECIS system converted the impedance data to resistance and capacitance of the cell monolayer. These represent barrier function and membrane capacitance, respectively. Transendothelial resistance (TER) is presented as an index of endothelial barrier function.

### Determination of Permeability of Endothelial Cell Monolayers

The apparent permeability coefficients of albumin (*P*_s_^albumin^) of HUVEC monolayers were determined using our previously described protocol, with minor modifications [3]. Cells were transfected as described above, and were seeded at a density of 1.5 x 10^5^ cells onto individual gelatin-coated Costar Transwell membranes (no. 3470, 0.4 μm pores, VWR, Houston, TX) and allowed to form a monolayer overnight. Medium was changed to phenol-red-free EBM (Lonza) for 2 h prior to the experiment. AlexaFluor-488-albumin was added to the luminal (upper) chamber to a final concentration of 1 mg/ml. After 1 h, samples were obtained from the luminal and abluminal (lower) chambers, and the fluorescence intensities were measured with a SpectraMax M3 plate reader (Molecular Devices, Sunnyvale, CA) and albumin concentrations determined using a standard curve. *P*_s_^albumin^ was calculated as *P*_s_^albumin^ = [A]/*t* x 1/*A* x v/[L]; where [A] is the abluminal albumin concentration, *t* is time in seconds, *A* is the area of the membrane in cm^2^, V is the volume of the abluminal chamber, and [L] is the luminal albumin concentration.

### Determination of Permeability in Isolated Rat Mesenteric Venules

Male Sprague-Dawley rats (280–320 g) were housed in a controlled temperature (22°C) and controlled illumination (12:12 h light dark cycle) environment. After arrival, the rats were submitted to a one-week acclimation period and were provided standard rat chow (2018 Teklad Global 18% Protein Rodent Diet, Harlan) and water *ad libitum*. A total of n = 17 rats were used in this study. For venule isolation, each rat was anesthetized with ketamine/xylazine (90/9 mg/kg i.m.), a midline laparotomy was performed, and the small intestine and mesentery were exteriorized, excised, and placed in ice-cold APSS. The rats were euthanized with an overdose of ketamine/xylazine, confirmed by opening the chest. The lower ileum and associated mesentery were pinned in a dissection chamber containing ice-cold APSS, and a mesenteric venule (40–80 μm diameter, 0.5–1.0 mm length) was carefully dissected, excised and transferred to an isolated vessel chamber containing APSS. The venule was cannulated on each end with resistance-matched inflow and outflow micropipettes, with a third, smaller pipette inserted concentrically in the inflow pipette as previously described [3,38]. Each micropipette was connected to an adjustable reservoir to allow independent control of intraluminal pressure and flow. The venule was interchangeably perfused with either APSS from the outer inflow pipette or APSS containing Alexa Fluor 488-albumin from the inner pipette. Venular permeability was quantified by measuring the ratio of transvascular flux to the transmural concentration difference of the tracer [38]. *P*_s_^albumin^ was calculated as *P*_s_^albumin^ = (1/ΔI_f_)(dI_f_/dt)_0_(r/2), where ΔI_f_ is the initial step increase in fluorescent intensity when switching to APSS containing Alexa Fluor 488-albumin, (dI_f_/dt)_0_ is the initial rate of gradual increase in intensity as the solute tracer diffuses out of the vessel, and r is the venular radius. In each experiment venules were perfused at a constant perfusion pressure of 10 cm H_2_O at a flow velocity of 7 mm/s [38].

### MLC Phosphorylation Studies

Confocal microscopic imaging of dually phosphorylated MLC (on Thr18 and Ser19) was used to determine the degree and localization of MLCK activation in cultured HUVEC. Cells grown on coverslips were fixed in 4% paraformaldehyde and permeabilized with 0.1% Triton X-100. Blocking solution (5% donkey serum) was applied for 1 h, and rabbit anti-phospho-MLC2-T18/S19 (1:200) and goat anti-VE-cadherin (1:50) were applied overnight at 4°C. After three 5-min washes, AlexaFluor 488-donkey anti-rabbit IgG (1:100) and Alexa Fluor-647-donkey anti-goat (1:100) antibodies were applied for 1 h. F-actin was labeled by a 30-min incubation with Texas Red-phalloidin. The coverslips were mounted onto slides with Prolong Gold anti-fade reagent with DAPI, and sealed with nail polish. Confocal image z-stacks were acquired at the USF Lisa Muma Weitz Laboratory for Advanced Microscopy and Cell Imaging, with an Olympus FV1000 microscope system using a 60X oil immersion objective and FV10-ASW version 3.0 software (Olympus America, Center Valley, PA). Up to 8 optical z-sections were acquired at 1-μm intervals. Maximum intensity z-projections are presented.

### Rho family GTPase Activation Assays

Colorimetric G-LISA activity assay kits (Cytoskeleton, Inc., Denver, CO, catalog numbers BK124, BK127, and BK128) were used according to the manufacturer’s instructions to quantitatively assess GTP-bound RhoA, Rac1, and Cdc42 levels in HUVEC. Cells were grown to confluence in gelatin-coated 100 mm culture dishes, and the medium was changed to serum-free EBM the day before the experiment. After experimental treatments, the cells were washed with ice-cold (4°C) PBS and then lysed in ice-cold lysis buffer. The lysate was clarified at 14000 x g at 4°C for 2 min, a 20 μl aliquot was taken for a protein assay, and the remaining lysate was separated into at least two aliquots, snap frozen in liquid nitrogen, and stored at −70°C until the start of the ELISA portion of the assay. Protein concentrations were determined using the Precision Red Advanced Protein Assay that came with the kits. Snap frozen lysate was then thawed and the sample protein concentrations were equilibrated using lysis buffer. GTP-bound RhoA, Rac1, or Cdc42 levels were then determined using the RhoA-GTP, Rac1-GTP, Cdc42-GTP binding 96-well plates, including a lysis buffer blank control and GTP-bound recombinant positive controls (80 pg/ml). Absorption of the ELISA wells was determined with a Tecan Infinite 200 Microplate Reader (Tecan, Männedorf, Switzerland). We also routinely verified that the total amount of RhoA, Rac1, and Cdc42 (GTP- and GDP-bound) were equivalent between groups by Western blotting (data not shown).

### Data Analysis

Data are presented as mean ± SE. For comparisons of two groups, t-tests assuming equal variances were used. For two paired groups, we used paired t-tests. To compare 3 or more time points in a time course study, repeated measures one-way ANOVA was used, followed by Dunnett’s test for post-hoc comparisons when appropriate. When multiple groups were compared over time, repeated measures two-way ANOVA followed by Bonferroni t-tests were used. For comparisons of three or more independent groups with only one time point measured, one-way ANOVA was used, followed by Tukey’s test for post-hoc comparisons when appropriate. Significance was accepted at P<0.05.

## Results

### Expression of GFP-Actin in HUVEC

GFP-actin expression was typically observed in >50% of transfected cells, with fields of view showing >95% expression often apparent (S3A Fig.). Compared to native β-actin, the relative amount of GFP-actin was very small (S3B Fig.). Fibers containing GFP-actin colocalized with F-actin labeled with AlexaFluor594-phalloidin (S3C-E Fig.). These data confirm that GFP-actin and native β-actin monomers combine to form actin fibers in the transfected HUVEC.

### GFP-Actin Expression and HUVEC Barrier Function

We evaluated whether GFP-actin expression might alter the barrier function of cultured HUVEC monolayers, and found no significant difference between the baseline TER of untransfected HUVEC and HUVEC expressing GFP-actin (10037 ± 631 vs. 9094 ± 257 Ω, respectively). We also saw no difference in the reduction in TER caused by thrombin between these two groups (S3F Fig.), indicating that GFP-actin expression has no effect on baseline endothelial barrier function or thrombin-induced barrier dysfunction.

### Actin Cytoskeleton Dynamics in HUVEC

Dynamics of actin fibers before and after thrombin challenge were studied using time lapse imaging of GFP-actin expressed in confluent HUVEC monolayers. Cells were typically stationary, and motile cells with a well-defined leading edge were rare. Interestingly, in stationary cells, there was an ongoing formation and withdrawal of actin-rich protrusions, mainly local lamellipodia around the entire perimeter of most cells (S1 Movie). We also observed dynamic actin ring structures that expanded concentrically, previously named actin clouds, which when near the edge of a cell gave rise to lamellipodia [31]. Most of the longer actin fibers were cortical actin fibers near the intercellular junctions.

### Impact of Thrombin on Actin Cytoskeleton Dynamics in HUVEC

**Fig 1:**
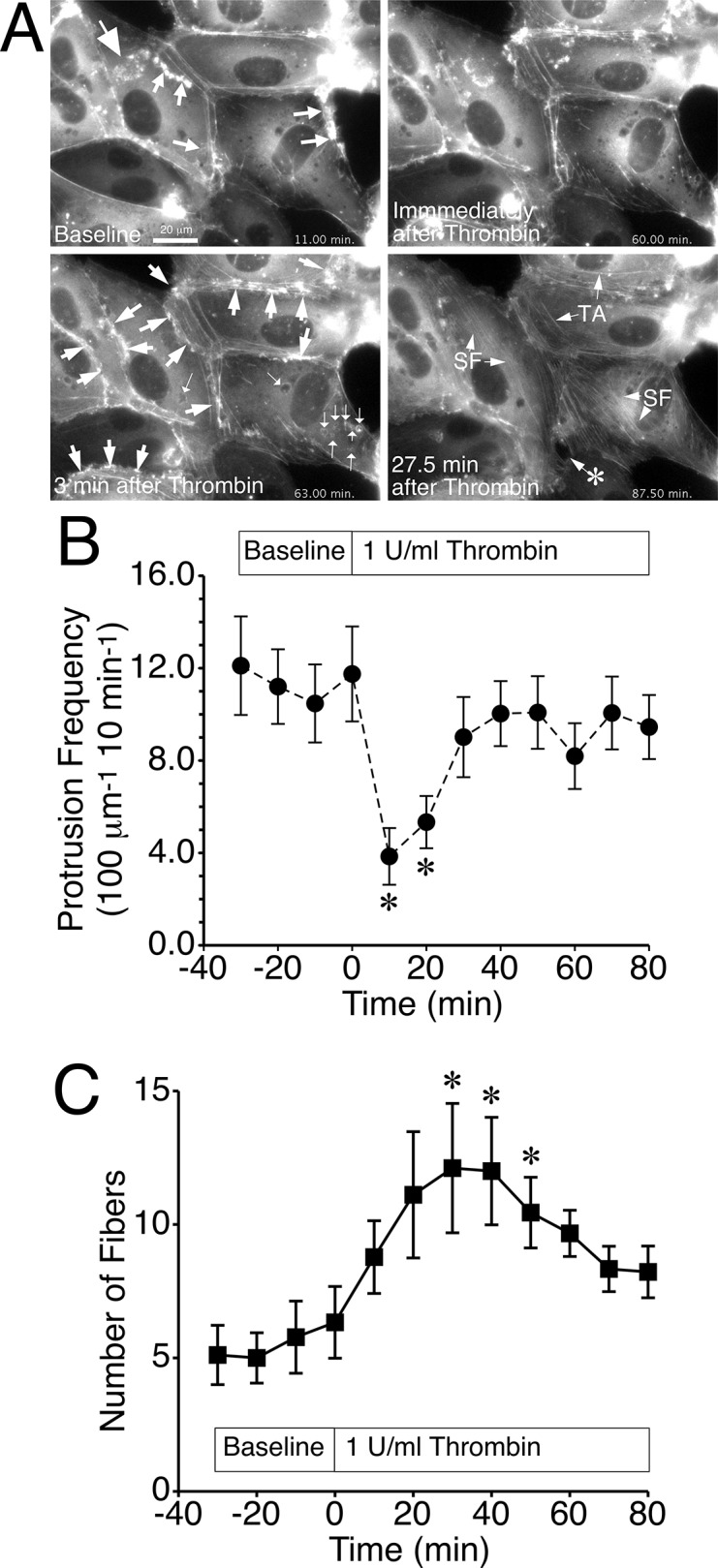
Thrombin-induced decreased lamellipodia protrusion frequency and actin stress fiber formation. *A*. HUVEC expressing GFP-actin displayed frequent formation and withdrawal of local lamellipodia (small arrows) and actin “clouds” (large arrowhead) during baseline recording. Shortly after addition of thrombin (1 U/ml; 3 min image) there was a coordinated increase in actin at the periphery of cells (thin arrowheads) and around vesicles (very small arrows) followed by a sharp decline in protrusive activity. Later (27.5 min image), ventral stress fibers (SF) formed de novo in the cells, and cortical actin migrated to become transverse arc (TA) stress fibers. Also, in this image a transient opening at a tricellular junction (*) is apparent. *B*. Thrombin initially decreased the mean protrusion frequency of local lamellipodia. *C*. The number of actin fibers significantly increased at 30 min after the addition of 1 U/ml thrombin. *P<0.05 versus the zero-minute time point. N = 9 cells studied in the imaging experiments.

When thrombin was added to the cells, there was a brief increase in GFP-actin at the cell periphery and on some vesicles, followed by a redistribution of actin throughout the cytoplasm, a loss of actin cloud and lamellipodia formation, slight changes in the shapes of cells, and the formation of transient gaps between cells (S2 Movie and Fig. 1A). Thrombin significantly decreased the protrusion frequency of local lamellipodia for up to 20 min (Fig. 1B). In this experiment, we also observed a significant decrease in protrusion velocity at the 20 min time point, and a significant increase in withdrawal time at the 10 min time point, but no other significant changes in other measures of lamellipodia protrusion or withdrawal over the time course (S4 Fig.). The number of actin stress fibers significantly increased at the 30, 40, and 50 min time points (Fig. 1C). Two apparent, independent mechanisms accounted for the increase. First, cortical actin fibers moved toward the center of the cell, becoming stress fibers, and resembling transverse arcs, a subset of stress fibers observed in migrating cells (S2 Movie). Under baseline conditions, the mean velocity of these fibers toward the center of the cell was approximately 0.1 μm/min When thrombin was added, the velocity of lateral displacement of actin stress fibers toward the center of the cell increased two-fold within the first 10 min after thrombin was added (S5 Fig.). The second mechanism for stress fiber formation was assembly/bundling of new actin stress fibers and the extension of smaller, preexisting fibers in the central area of the cell (S2 Movie, large arrowheads). The fibers generated by this mechanism best fit the description for the subset of stress fibers referred to as ventral stress fibers [39,40]. Of these different classifications, the transverse arcs appeared to be more stable structures of the two subsets, as the ventral stress fibers began disassembling 30–40 min after thrombin was added.

The other notable feature following thrombin treatment was the formation of small gaps between cells, such as the one seen at the junction of three cells shown in S2 Movie and Fig. 1A. Taking a closer look at the formation and turnover of this gap (S3 Movie), we observed that a sudden recoil of pre-existing cortical actin fibers that terminated at the junctions between cells was associated with the retraction of cells. Shortly after, lamellipodia filled the space between cells until the gap was closed. After closure, many small local protrusions formed over the filled space, suggesting a potential role for local lamellipodia to help repair broken junctions when endothelial barrier integrity is compromised.

### Impact of S1P on Actin Cytoskeleton Dynamics in HUVEC

**Fig 2:**
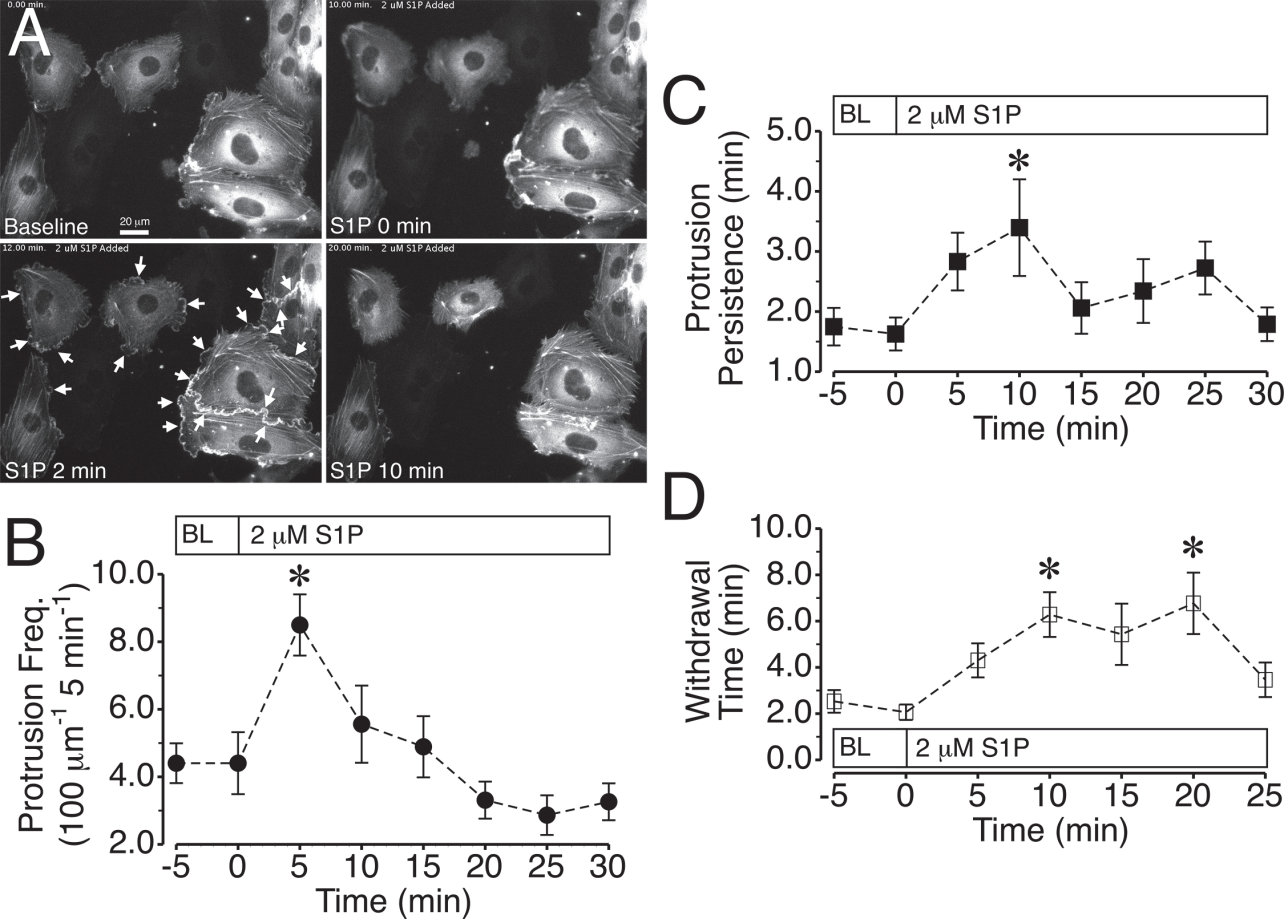
S1P causes longer-lasting lamellipodia protrusions. *A*. HUVEC expressing GFP-actin displayed frequent protrusion and withdrawal of lamellipodia, (baseline-0 min S1P) and addition of 2 μM S1P caused a coordinated increase in protrusion of lamellipodia (2 min, arrows). Within 10 min, the initial lamellipodia that had formed after S1P was added typically had withdrawn. *B*. S1P caused a brief, significant increase in protrusion frequency. *C*. Protrusion persistence also increased significantly at 10 min after S1P was added *D*. Withdrawal time was significantly sustained for at 10 and 20 min after S1P was added. *P<0.05 versus time 0 min (baseline). For the imaging experiments, N = 9 cells were studied.

We next tested whether adding an agent that enhances endothelial barrier integrity might impact local lamellipodia in endothelial cells in a roughly opposite fashion as thrombin. We utilized S1P, a physiologically relevant bioactive lipid that reduces permeability *in vivo* and *in vitro* [41–44]. We verified that GFP-actin expression in HUVEC did not impact the ability of S1P to increase TER compared to mock-transfected cells (S3G Fig.). Addition of 2 μM S1P caused a rapid and coordinated increase in protrusion all along the edges of endothelial cells (S4 Movie), with many new local lamellipodia evident within 2 min after S1P was added (Fig. 2A). The summarized data show that protrusion significantly increased within 5 minutes of the addition of S1P, however quickly returned towards baseline after the 5-minute time point (Fig. 2B). Protrusion distance and velocity were not significantly altered by S1P treatment (S6A, B Fig.), however mean protrusion persistence, which indicates the average time for lamellipodia to continue their spreading, was significantly elevated at the 10-minute time point (Fig. 2C). Although withdrawal distance and velocity were not affected (S6C, D Fig.), S1P significantly elevated the number of lamellipodia with withdrawal times greater than 5 min, which occurs when lamellipodia withdraw more slowly or produce a net gain in surface area covered (S6E Fig.). Withdrawal time was significantly elevated compared to baseline, approximately tripled at 10 and 20 min after S1P was added (Fig. 2D). Combined, these results indicate that the rise in TER during the first five minutes after S1P addition coincides with a rise in lamellipodia protrusion frequency, and that the elevated TER that persists thereafter is associated with decreased withdrawal of local lamellipodia.

### Impact of S1P on Thrombin-Induced Endothelial Barrier Dysfunction

**Fig 3:**
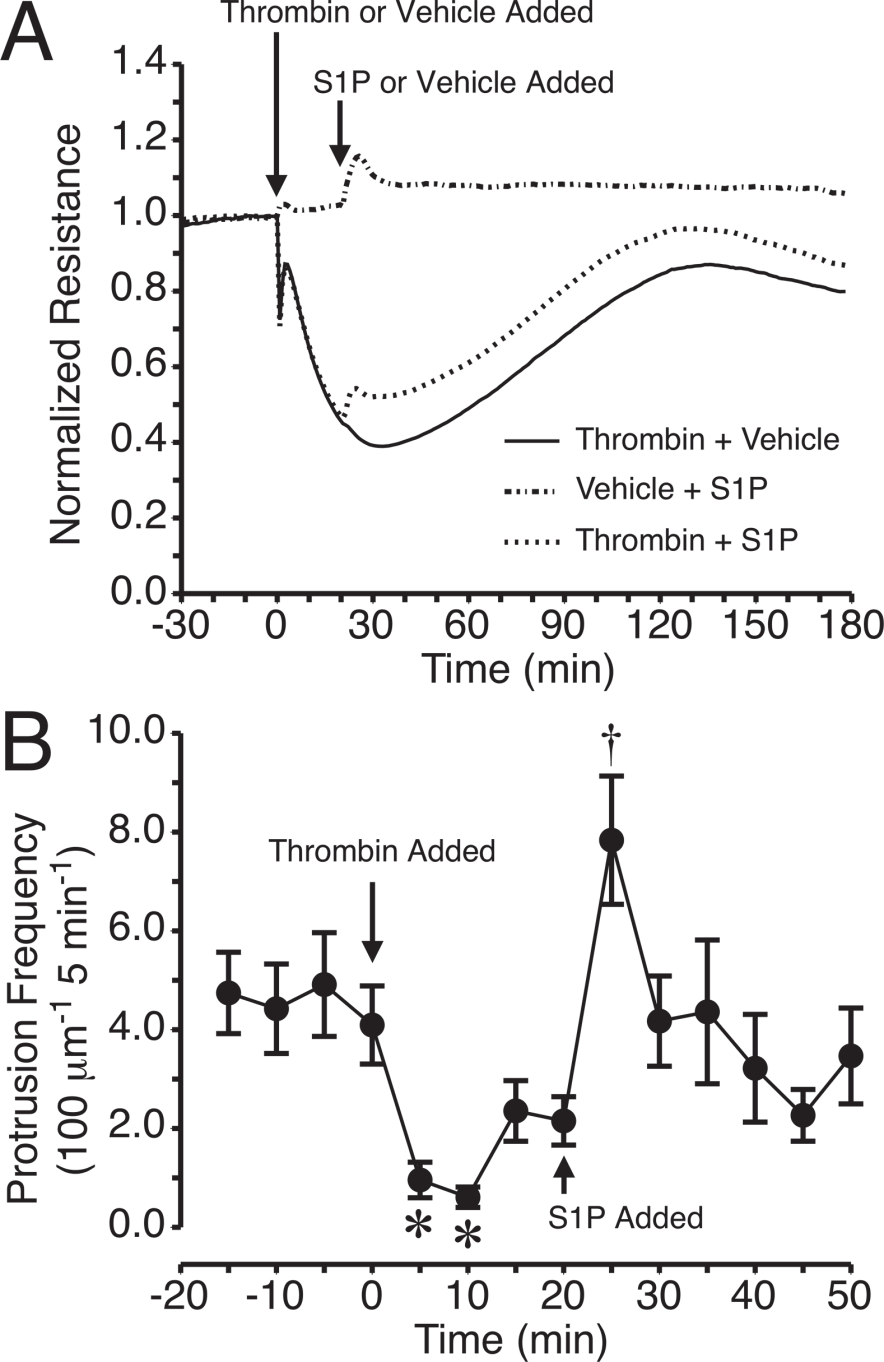
Impact of S1P during thrombin-induced endothelial barrier dysfunction. *A*. Time course of changes in TER of HUVEC monolayers treated with 1 U/ml thrombin or vehicle at the indicated time point, followed by addition of 2 μM S1P or vehicle 20 min. later. The TER tracings are an average for N = 8 electrode wells in each group. *B*. Protrusion frequency of HUVEC expressing GFP-actin treated with 1 U/ml thrombin, followed by 2 μM S1P 20 min later. *P<0.05 versus the 0 min time point when thrombin was added. †P<0.05 versus the 20 min time point when S1P was added. N = 9 cells studied.

We tested whether S1P may ameliorate thrombin-induced endothelial barrier dysfunction with a protocol in which HUVEC monolayers were first treated with 1 U/ml thrombin, and 20 min later 2 μM S1P was added. This group was compared to cells that were treated with only thrombin or S1P. After thrombin caused a decrease in TER, the addition of S1P caused a slight elevation in TER over the remainder of the time course compared to thrombin alone (Fig. 3A). The elevation in TER elicited by S1P in the presence of thrombin was roughly the same magnitude as for S1P alone in the absence of thrombin. The same protocol was also tested using HUVEC expressing GFP-actin (S5 Movie). In a similar fashion as in Fig. 1, thrombin decreased the lamellipodia protrusion frequency (Fig. 3B). After the addition of S1P, protrusion frequency significantly increased within 5 min (Fig. 3B). Withdrawal time also significantly increased 15–20 min after the addition of S1P, while other parameters were not significantly changed (S7 Fig.). The increases in protrusion frequency and withdrawal time were similar to those observed with S1P alone (Fig. 2). These data provided additional support that changes in lamellipodia protrusion frequency may directly correlate with changes in endothelial barrier function.

### Local Lamellipodia and Junctions Between Endothelial Cells

**Fig 4:**
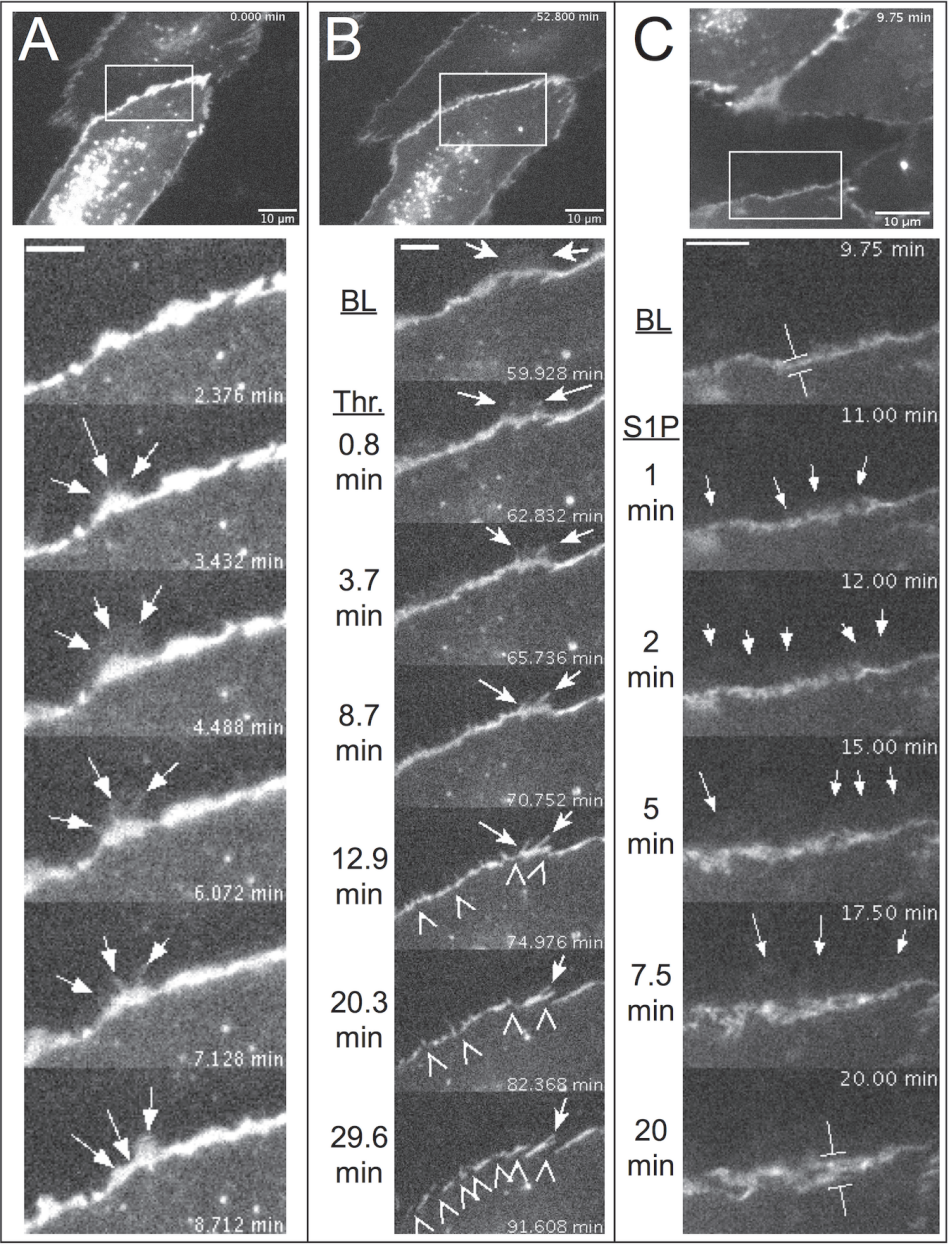
Local lamellipodia protruded beyond endothelial adherens junctions containing VE-cadherin-GFP and were associated with junction stability. At the top of all three panels, an image of HUVEC expressing VE-cadherin-GFP is shown. The bounding box in each top image shows the area studied in the time-lapse montages below. Confluent monolayers were used for all experiments, but not all cells expressed detectable levels of VE-cadherin-GFP. *A*. Time-lapse imaging revealed that VE-cadherin-GFP was most intense at intercellular junctions and in vesicles around the nucleus. Select time-lapse images (from S6 Movie) of the area in the box from top panel show the protrusion and withdrawal of a local lamellipodium (arrows) that spread toward the cell in the top of the image from the belt of VE-cadherin-GFP located between two cells. *B*. The same cells were tracked just before and during 1 U/ml thrombin treatment. Selected time-lapse images from the bounding box in the top panel (from S7 Movie) show how the withdrawal of a local lamellipodium that had protruded prior to thrombin treatment yielded filopodia-like structures containing VE-cadherin (arrows). Subsequently, as fewer lamellipodia protruded from the cell edge, breaks in the continuous belt of VE-cadherin emerged (arrowheads). *C*. Time-lapse studies before and after treatment with 2 μM S1P (from S8 Movie) show that lamellipodia spread beyond the VE-cadherin-GFP-rich junctions (arrows). In addition, over time the junctional areas containing VE-cadherin-GFP appeared wider than during baseline (compare the calipers at BL and 20 min). Images are representative of observations from at least three different experiments each with thrombin and S1P.

Because endothelial cell-cell junctions have a key role in regulating paracellular transport [1,45], we investigated the dynamics of the endothelial junction protein VE-cadherin using confluent HUVEC expressing a VE-cadherin-GFP fusion protein. VE-cadherin-GFP was detected predominantly at the cell periphery in a similar fashion as previously reported for native VE-cadherin by immunofluorescent labeling [46,47], and also was very intense in vesicles around the nucleus (S6 Movie). In addition, enough of the expressed VE-cadherin-GFP was located ubiquitously to detect an entire cell’s footprint. This enabled observation of numerous lamellipodia protruding beyond the belt of VE-cadherin-GFP located at intercellular junctions, causing transient overlapping with adjacent cells (Fig. 4A). Most lamellipodia did not appear to have high amounts of VE-cadherin-GFP, although occasionally there were exceptions, such as the newly forming lamellipodium in the last image of the montage in Fig. 4A. Compared to local lamellipodia, most of the belt of VE-cadherin-GFP at endothelial cell-cell junctions was very stable and its movements were relatively slow.

After treatment with 1 U/ml thrombin, there was an apparent decrease in lamellipodia, followed by the transformation of the continuous VE-cadherin-GFP belt into a discontinuous pattern (S7 Movie). Also, in places where a lamellipodium had been located just prior to thrombin treatment, we noticed that VE-cadherin-GFP-containing structures that resemble filopodia often remained (Fig. 4B). In contrast to thrombin treatment, S1P caused more lamellipodia to form, and these protruded well beyond the VE-cadherin-GFP belt at junctions (S8 Movie and Fig. 4C). We also observed a progressive widening of the VE-cadherin-GFP belt after S1P was added (Fig. 4C) Taken together, these data further support the association between local lamellipodia and endothelial barrier function.

### Inhibition of myosin II attenuates local lamellipodia formation and increases endothelial permeability

**Fig 5:**
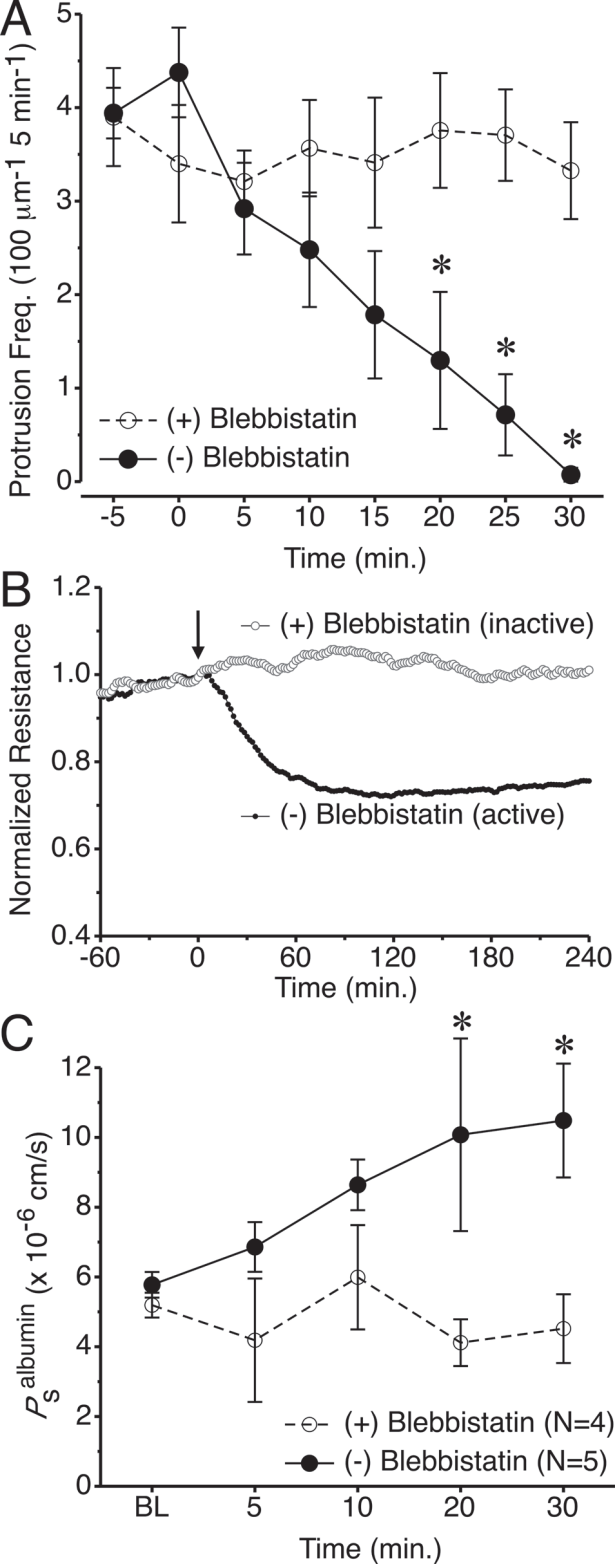
Impact of the myosin II inhibitor blebbistatin on endothelial lamellipodia and barrier function. *A*. Local lamellipodia protrusion frequency in HUVEC expressing GFP-actin. At time = 0 min, cells were treated with 100 μM of (-)blebbistatin, or the inactive control, (+)blebbistatin. *P<0.05 between groups, same time point. N = 9 cells per group. *B*. Changes in TER in response to 100 μM (-) or (+)blebbistatin, added at time = 0 min (arrow). N = 4 each group. *C*. Time course of changes in *P*_s_^albumin^ in response to 100 μM (-) or (+)blebbistatin. *P<0.05 between groups, same time point.

We sought a strategy to more selectively inhibit local lamellipodia in endothelial cells that could be used to test their putative role in maintenance of endothelial barrier function. We found the myosin II inhibitor, blebbistatin, to be a good candidate for this purpose. Application of its active enantiomer, (-)blebbistatin, steadily decreased the lamellipodia protrusion frequency in HUVEC expressing GFP-actin over a 30-min period, with little apparent impact on cortical actin fibers or stress fibers (S9 Movie and Fig. 5A). Analysis of the time-lapse images revealed that (-)blebbistatin also significantly reduced protrusion distance at 15, 25, and 30 min, but did not change any other protrusion or withdrawal parameters (S8 Fig.). In contrast, the inactive enantiomer, (+)blebbistatin had no impact on lamellipodia protrusion/withdrawal (S9 Movie, Fig. 5A, and S9 Fig.).

In studies of endothelial barrier function, (-)blebbistatin steadily reduced TER approximately 25% over a 1 h period, after which a new steady-state TER developed, while the inactive enantiomer, (+)blebbistatin, caused no change in TER from baseline (Fig. 5B). To evaluate whether this finding applied to intact vessels, we applied 100 μM (-)blebbistatin to isolated, perfused venules, and found that it significantly increased permeability. In contrast, 100 μM (+)blebbistatin caused no change in permeability (Fig. 5C). While there may be some limitations with this approach, as (-)blebbistatin does globally affect actin-myosin mediated contraction, these data show that myosin II activity contributes to local lamellipodia formation, and that loss of local lamellipodia is detrimental to normal endothelial barrier integrity.

### Thrombin and S1P both cause phosphorylation of MLC

**Fig 6:**
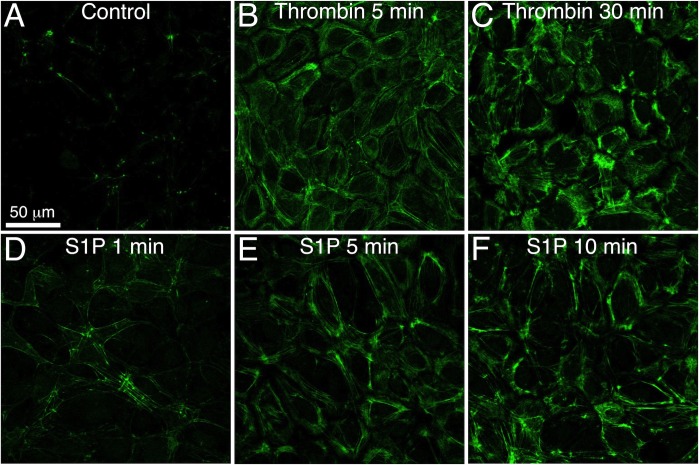
Thrombin and S1P increase phosphorylation of MLC on Thr-18/Ser-19. Confocal images of immunofluorescence labeling of dually phosphorylated myosin within HUVEC monolayers are shown. The cells were either untreated controls (*A*) or treated with 1 U/ml thrombin (*B*, *C*) or 2 μM S1P (*D*, *E*, *F*) for the durations indicated in each panel. Scale bar = 50 μm. Representative of three separate experiments.

Increased phosphorylation of MLC has previously been reported to contribute to microvascular hyperpermeability in response to certain inflammatory stimuli [11,48], but also may participate in barrier enhancement [49]. Thus, we evaluated whether the differential impacts of thrombin and S1P on endothelial barrier integrity may be due to their impacts on MLC phosphorylation, particularly localization. We found, however, that both thrombin and S1P increased phosphorylation of MLC on T18/S19 (Fig. 6). For both thrombin and S1P, most of the phosphorylated MLC localized on actin fibers. These were mostly the cortical actin fibers parallel to edges of cells, although thrombin also caused some appearance of stress fibers as well (Fig. 6). This was confirmed with co-labeling using Texas-Red phalloidin (data not shown). From these data there does not appear to be a correlation between MLC phosphorylation and barrier function.

### Rho family GTPase activity after thrombin or S1P

**Fig 7:**
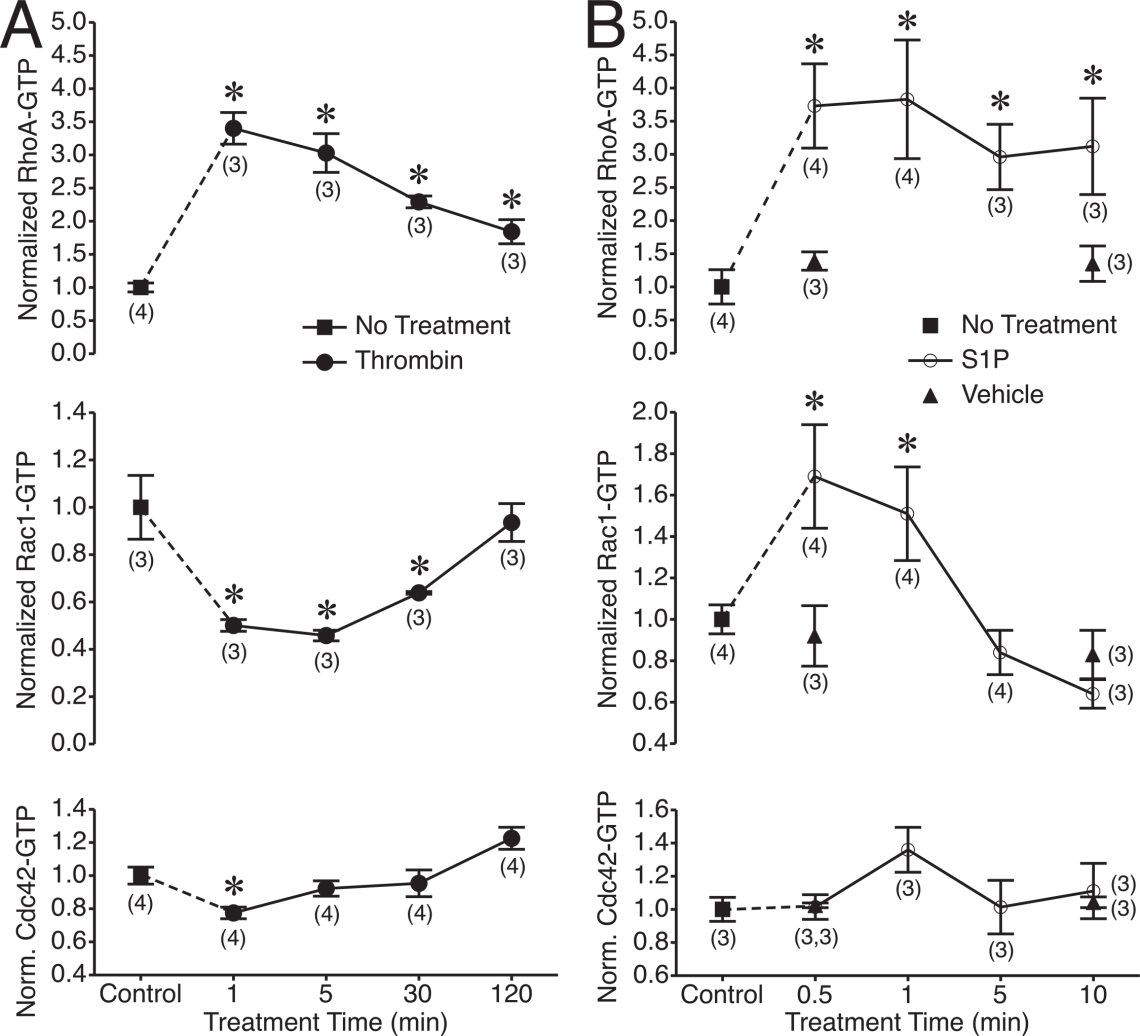
GTP-bound RhoA, Rac1, and Cdc42 levels in response to thrombin and S1P. *A*. Impact of thrombin on GTP-bound RhoA, Rac1, and Cdc42 in cultured HUVEC. Untreated cells served as control. *B*. Impact of S1P on GTP-bound RhoA, Rac1, and Cdc42 in cultured HUVEC. Untreated cells served as control, and vehicle controls were also tested at the 0.5-min and 10-min time points. The numbers in parentheses indicate the number of replicates for each group. *P<0.05 compared to control (no treatment).

Given their reported roles in modulation of lamellipodia and endothelial permeability, we examined the activation of the Rho family small GTPases RhoA, Rac1, and Cdc42 in cultured endothelial cells treated with thrombin or S1P (Fig. 7). Within 1 minute, thrombin caused a 3.5-fold increase in GTP-bound RhoA concurrent with a significant reduction in Rac1-GTP (Fig. 7A). The RhoA-GTP levels remained significantly elevated for all time points in the 120-min time course, while Rac1-GTP levels remained significantly decreased from control through the 30-min time point. Thrombin also significantly decreased Cdc42-GTP levels at the 1-min time point (Fig. 7A). Treatment with S1P increased RhoA-GTP levels 3.5-fold within 30 sec, and RhoA-GTP remained significantly elevated compared to control during the remainder of the 10-min time course (Fig. 7B). S1P also significantly elevated Rac1-GTP levels at the 30 sec and 1 min time points, while having no significant impact on Cdc42-GTP levels (Fig. 7B).

### Inhibition of Rac1 reduces frequency local lamellipodia and increases endothelial permeability

**Fig 8:**
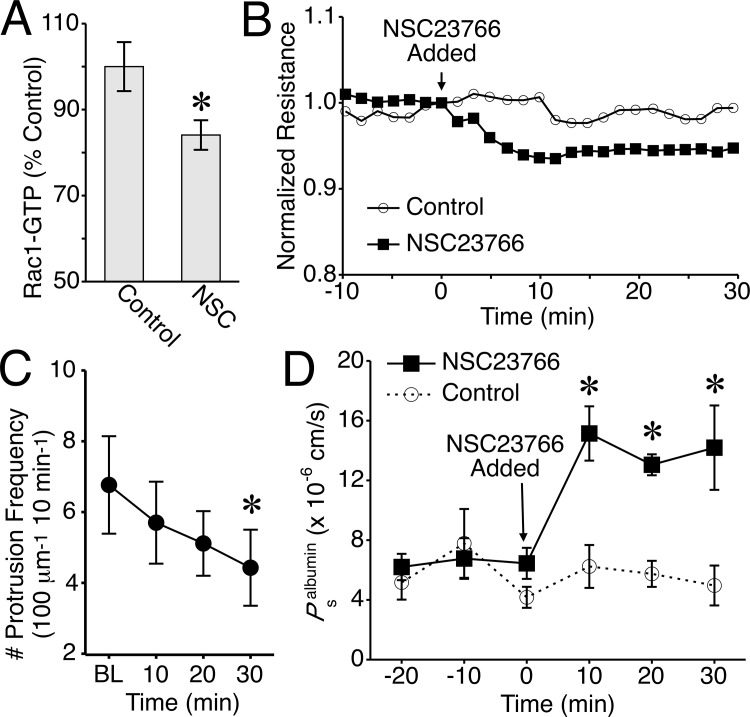
Inhibition of Rac1 decreases lamellipodia formation and increases endothelial permeability. *A*. Rac1-GTP levels in control HUVEC and cells treated with 50 μM NSC23766 for 30 min. *B*. Time course of mean changes in TER caused by 50 μM NSC23766 (N = 8), compared to control (N = 8). *C*. Time course of changes in lamellipodia protrusion frequency after the addition of 50 μM NSC23766 (N = 9 cells studied. *P<0.05 versus baseline (BL). *D*. Time course of changes in the permeability of isolated rat mesenteric venules to albumin in response to 50 μM NSC23766 (N = 4) compared to control (N = 4). *P<0.05 versus control, same time point.

Because thrombin and S1P caused changes in Rac1-GTP that correlated with both barrier function and lamellipodia protrusion frequency, we chose to focus our investigation on Rac1. We used the pharmacologic inhibitor NSC23766 to test its role. This compound inhibits the activation of Rac1 by the guanine exchange factor Tiam1, which has previously been implicated in promoting endothelial barrier integrity [19,50]. While concentrations of 200 μM NSC23766 have been reported to disrupt barrier integrity of human dermal endothelial cells and mouse myocardial microvascular endothelial cells [26,51,52], we elected to use a slightly lower concentration (50 μM) out of concerns for specificity and selectivity. Application of NSC23766 at 50 μM has previously shown to decrease Rac1-GTP in NIH 3T3 cells [53] and also to decrease barrier function of bovine brain microvascular endothelial cells [54] and adult human dermal lymphatic endothelial cells [27]. In the current study, 50 μM NSC23766 decreased Rac1-GTP levels, TER, and lamellipodia protrusion frequency (Fig. 8A-C and S10 Movie). While the NSC23766-induced decreases in these parameters were relatively subtle compared to those elicited by thrombin (Figs. 1 and 3), the decreases in Rac1-GTP and protrusion frequency were significant. Other protrusion/withdrawal parameters measured did not show any significant changes (S10 Fig.). To determine whether the barrier function observations extend to microvessels, we tested the impact of 50 μM NSC23766 on isolated rat mesenteric venules (Fig. 8D), and observed a two-fold increase in the permeability to albumin that was sustained for up to 30 min.

**Fig 9:**
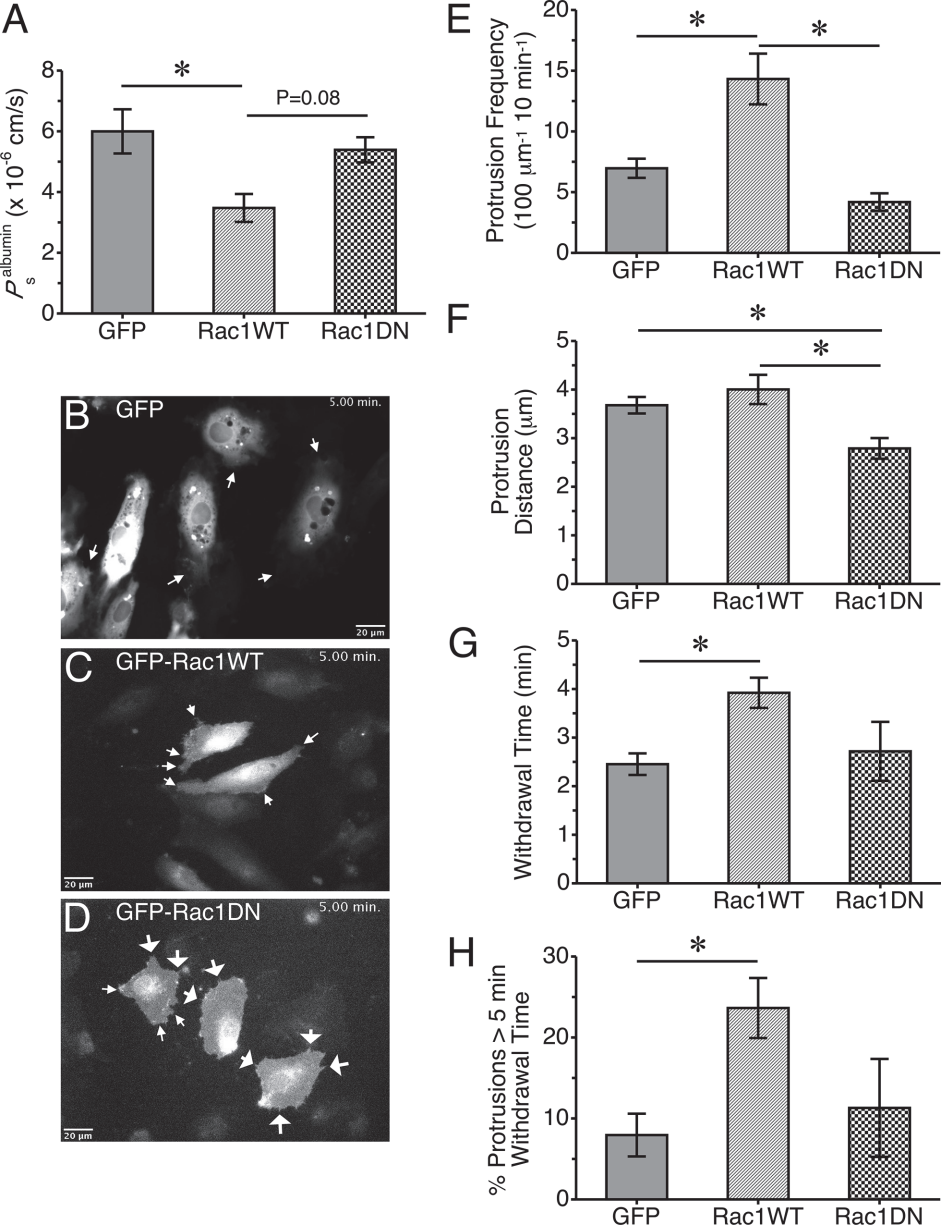
Impact of overexpression of wild-type (WT) or dominant-negative (DN) Rac1 on endothelial barrier function and local lamellipodia dynamics. *A*. *P*_s_^albumin^ of HUVEC monolayers expressing GFP, GFP-Rac1-WT, or GFP-Rac1-DN (N = 4 for each group) ~16 h after transfection. Panels *B*, *C*, and *D* show expression of each construct, also shown in S11 Movie. These images were obtained ~16 h after transfection. The small arrows indicate lamellipodia, while the arrows with wider arrowheads show filopodia that were prevalent in cells expressing GFP-Rac1-DN. Lamellipodia parameters were also evaluated over a 10-min period: *E*. Protrusion frequency, *F*. Protrusion distance, *G*. Withdrawal Time, *H*. %Protrusions with a withdrawal time > 5 min. *P<0.05 between the indicated groups. N = 9 cells studied in each group.

In addition to the pharmacologic inhibition studies, we also used an overexpression approach. Transfection of HUVEC with plasmids encoding a GFP-Rac1 fusion protein, resulting in an overexpression of Rac1, decreased solute permeability (Fig. 9A) compared to HUVEC expressing GFP alone. In contrast, when HUVEC expressed a dominant negative form of Rac1, GFP-Rac1T17N, the cell monolayers had no significant change in solute permeability (Fig. 9A) compared to the GFP control. Local lamellipodia were quite apparent in HUVEC transfected with GFP or GFP-Rac1, however in cells expressing GFP-Rac1T17N, filopodia were more common than lamellipodia (S11 Movie and Fig. 9B, C, D). Expression of GFP-Rac1 in HUVEC caused an increase in local lamellipodia protrusion frequency and withdrawal time, compared to cells expressing GFP (Fig. 9E-H). Expression of GFP-Rac1T17N caused a slight decrease in lamellipodia protrusion frequency, a significant decrease in protrusion distance, and no impact on withdrawal time compared to cells expressing GFP (Fig. 9E-H). Other protrusion/withdrawal parameters were not significantly changed by expression of GFP-Rac1 or GFP-Rac1T17N, compared to GFP expression (S11 Fig.). Withdrawal distance was significantly different between the GFP-Rac1 and GFP-Rac1T17N groups (S11 Fig.), in a similar fashion as the protrusion distance (Fig. 9F).

## Discussion

The importance of junctional protein complexes, such as those composed of VE-cadherin and its associated catenins have been well established in the control of microvascular permeability [1,2]. However, a more detailed understanding of the time course of the cytoskeletal and junctional mechanisms elicited by agents that alter endothelial barrier function requires the ability to more precisely view changes in these subcellular structures in living endothelial cells. Our development of a time-lapse imaging protocol using HUVEC expressing GFP-actin led to the initial observations that local lamellipodia are prevalent in confluent endothelial cell monolayers [32]. The current study combined these fluorescent time-lapse imaging protocols with techniques to precisely measure changes in endothelial barrier function over time.

Based on the following observations from our current data, we conclude that in addition to the relatively stable junctional adhesions containing VE-cadherin, local lamellipodia represent a more dynamic adhesive structure that contributes to endothelial barrier integrity. The following observations support this conclusion. First, under baseline conditions, local lamellipodia were observed to cause transient changes in overlap between adjacent cells, extending beyond the continuous band of VE-cadherin. Second, studies with thrombin and S1P revealed a correlation between changes in lamellipodia protrusion frequency and endothelial barrier function. Third, application of the myosin II inhibitor (-)blebbistatin decreased local lamellipodia formation without affecting actin fibers, and this correlated with decreased endothelial barrier function. Fourth, treatment with (-)blebbistatin increased the permeability of intact, perfused rat mesenteric venules, suggesting that the changes in barrier function observed in cell culture translate to intact microvessels. Lastly, we also reduced lamellipodia protrusion frequency and barrier function together by inhibiting Rac1, and produced opposite results when overexpressing Rac1. Taken together, these data support the concept that local lamellipodia are involved in the control of microvascular permeability. In addition, the current results are concordant with two recent reports. In the first, a study by Martinelli et al, similar structures referred to as ventral lamellipodia were shown to selectively close pores produced by leukocyte transmigration across the endothelium [55]. In a second report, Abu Taha et al described “junction-associated intermittent lamellipodia” which facilitated the strengthening of endothelial cell-cell contacts and were strongly influenced by the state of VE-cadherin binding between endothelial cells [56].

The lamellipodia protrusion velocity we observed was similar to that in previous studies using HUVEC [57] and B16 melanoma cells [58]. We did see some variation in responsiveness to thrombin and S1P between experiments. For example, the significant differences protrusion velocity and withdrawal time in response to thrombin observed in one set of experiments (S4 Fig.), did not manifest in a subsequent study (S7 Fig.). These differences may be due to biological variability. On the other hand, we observed very consistent changes in lamellipodia protrusion frequency in response to thrombin or S1P, so of the various parameters we measured, we consider this one to be of primary importance.

The molecular mechanisms underlying protrusion, pause, and withdrawal of lamellipodia have not been fully elucidated. However, an elegant study utilizing correlated live cell imaging and electron microscopy shows that actin filaments are organized in a meshwork with relatively steep angles to the cell edge and polymerize to push the cell edge forward during protrusion. During pause and withdrawal of lamellipodia, the angles of these filaments become shallower and may contribute to the formation of actin stress fibers [58]. The lamellipodia that contribute to the formation or restoration of contacts between adjacent endothelial cells were reported to require the Arp2/3 complex [55,56]. There may also be actin-myosin-independent mechanisms of cell spreading at this point, such as microtubule growth [59–61].

One previous report suggested that S1P-mediated cell spreading enhances endothelial barrier integrity independently of VE-cadherin [62]. We observed that local lamellipodia spread beyond the belt of VE-cadherin-GFP, which might provide some explanation to support this previous study. In the context of endothelial barrier function, local lamellipodia may contribute by increasing the ventral surface area covered by individual endothelial cells, causing adjacent cells to overlap more at junctions. This would result in a greater net diffusion distance via the paracellular pathway for solutes to cross the endothelial barrier. In this paradigm, additional homotypic binding of VE-cadherin on adjacent cells may not be required to reduce permeability, but a contribution of lamellipodia to facilitate VE-cadherin to the periphery is certainly not excluded, and is supported by recent reports [56,63].

Other insights arise from examination of the time course of thrombin-induced cytoskeletal changes and TER. The initial, rapid decrease in TER was previously shown to occur in the absence of MLC phosphorylation and actin-myosin-mediated contraction, and the MLCK inhibitor ML-7 failed to inhibit the initial drop [5,64,65]. Mathematical modeling of ECIS data acquired at multiple frequencies indicated that this initial decrease is predominantly due to opening of junctions, and to a lesser degree by a weakening of focal adhesions [5]. Concordant with these previous reports, in the current study the initial, rapid drop in TER was associated with the sudden movement of GFP-actin to intercellular junctions and to vesicles, and a transient cessation of local lamellipodia and actin cloud activity. There was also a slight change in the overall shapes of cells that might reflect a redistribution of tensile and supportive cytoskeletal elements [60]. While we did observe elevated phosphorylation of MLC within 5 min of thrombin treatment, we also observed it within 1 min of S1P treatment. Thus, MLC phosphorylation may be important for both barrier disruption and strengthening [11,48,49], or it may be an unrelated event for the initial stages. This represents a future area for additional investigation.

During the subsequent phase, when the TER continued to decrease gradually until reaching its nadir, some small gaps formed between cells, accompanied by a continued, significant decrease in lamellipodia protrusion frequency. The gap formation was associated with rapid centripetal retraction of the cell membrane, and this included rapid contraction of pre-existing cortical fibers, which appeared to be released from their focal or intercellular adhesions. This rapid movement probably reflects pre-stress generated by actin-myosin tension and possibly stretching of fibers that existed in the cell prior to the addition of thrombin [60,66,67]. Notably, the TER and lamellipodia protrusion frequency began to rise together toward baseline at the 30 min time point. At this stage, selective closing of gaps by local lamellipodia was also observed.

Unfortunately a limitation of our imaging of GFP-actin in live cells was that prolonged, repeated exposures to the excitation light often resulted in fading that reduced the usefulness of images after 2–2.5 hours. In addition, we had some concern that after repeated exposure to the excitation light there may be unidentified toxic effects [32]. Nevertheless, the key changes in the actin cytoskeleton in response to thrombin during the time frame studied that were identified improve understanding of the overall paracellular transport mechanism.

This study was also useful for identifying how actin stress fibers form in response to thrombin, and how these events relate temporally to previous measurements of thrombin-induced increases in isometric tension in endothelial cells. We observed two mechanisms of stress fiber formation in the current study. First, we observed that cortical actin fiber lateral migration to the center of the cell slightly accelerated. This mechanism reflected the previously described sub-type of actin stress fiber referred to as transverse arcs, often seen at within the lamella on the leading edge of a migrating cell [32,39,40]. The second type of actin stress fiber formation we observed were those where bundles assembled throughout the middle of the cytoplasm, gradually increasing in length, and eventually disassembling after a peak number of actin stress fibers was observed about 30 min following addition of thrombin (S2 Movie and Fig. 1D). This sub-population was very similar to ventral fibers in migrating cells [39,40], and were much more numerous than the transverse arcs. In addition, if they were indeed ventral stress fibers, they were likely connected to focal adhesions that would presumably contribute to the thrombin-induced increase in endothelial cell isometric tension [66,67]. Like the previously reported thrombin-induced isometric tension development, the appearance of actin stress fibers in endothelial cells lagged behind the initial decrease in TER [5,64].

The role of actin stress fibers in endothelial permeability is somewhat unclear. Stress fibers typically are observed after endothelial cells are treated with agents that increase permeability [1], and the tension they generate may prolong barrier dysfunction [5,64]. However, this is not always the case. In histamine-induced microvascular hyperpermeability, actin stress fibers were reported to form after barrier function had been restored to baseline [68]. In the current study, many of the new ventral stress fibers became visible after the cells had undergone a change in shape and some intercellular adhesions had already been disrupted, and these actin fibers gradually extended toward the cell periphery. It is conceivable that the appearance of actin stress fibers in endothelial cells may actually be part of a reflex response to disruption of intercellular or focal adhesions, similar to that seen in fibroblasts when a focal adhesion is experimentally disrupted [69]. Actin stress fibers are interesting structures in that they are rigid, with a load-bearing capacity, but at the same time due to both actin-myosin interactions and stretching by connections to focal or intercellular adhesions, produce isometric tension [66]. In endothelium where barrier function has been compromised, the increase in ventral stress fibers may serve to provide local shape stability due to their stiffness relative to the surrounding cytoplasm [67]. At the same time, however, the actin-myosin interaction within these stress fibers would produce increased isometric tension, which was previously proposed to inhibit the protrusive activities of the cells needed to re-establish junctional integrity [5,64].

Interestingly, (-)blebbistatin has been reported to drastically reduce endothelial cell isometric tension [70]. According to the cell tensegrity theory, several factors including centripetal pre-stress, centrifugal forces generated by microtubules, load-bearing properties of the cytoskeleton as a whole, and the connections between cytoskeletal elements and various adhesions at the cell membrane, provide the framework that maintain a stable cell shape in response to various physical forces [60]. The loss of lamellipodia and actin clouds caused by thrombin or (-)blebbistatin would result in a net loss of focal adhesions. Accordingly, we did see retraction of some cells in response to (-)blebbistatin treatment, probably reflecting some release of pre-stress within the elastic, cortical actin network. However, direct inhibition actin-myosin within stress fibers also needs to be accounted for, and this would also affect the overall tensegrity of the cell [60]. It is worth noting that in HUVEC expressing GFP-actin, we did not observe disappearance of actin stress fibers or cortical actin by (-)blebbistatin. This is in contrast to a previous report, however one major experimental difference was that the previous report used fixed, labeled cells [70]. We cannot discount that a significant decrease in isometric tension with (-)blebbistatin might alter cell shape in a way that contributes to endothelial barrier dysfunction. However, based on the facts that 1) actin fibers remained present in the cells, while 2) local lamellipodia nearly vanished, and 3) some retraction was seen in cells after local lamellipodia became less frequent, we think that the increased endothelial permeability caused by (-)blebbistatin is due to the impact on local lamellipodia.

Lastly, we investigated the potential roles of the Rho family GTPase RhoA, Rac1, and Cdc42, and observed a correlation between Rac1-GTP levels, protrusion frequency, and endothelial barrier function. Several other studies have shown that Rac1 contributes to endothelial barrier integrity [21–28]. In the current study, we present novel data demonstrating that Rac1 also promotes local lamellipodia protrusions in endothelial cells, which are differentially modulated by thrombin and S1P. One limitation with the G-LISA assays we used for detecting GTP-bound RhoA, Rac1, and Cdc42 is that they do not provide spatiotemporal data regarding activation. A recent study by Szulcek et al using a FRET biosensor for RhoA-GTP showed that spontaneous RhoA activity can be detected near cell borders in unstimulated endothelial cells. However, when thrombin is added, the RhoA activity becomes more centralized [71]. Thus, a future direction will are pursuing is the potential role of RhoA in lamellipodia formation using newer FRET biosensors.

In summary, the data support a role for Rac1-controlled, myosin II-mediated local lamellipodia in endothelial barrier integrity. Myosin II isoforms have recently been shown to have differential roles in lamellipodia formation [72] and represent a key future area for study. In addition, better understanding of the roles of microfilaments and intermediate filaments in lamellipodia formation and withdrawal may also help elucidate their roles in endothelial barrier function.

## Supporting Information

S1 Fig Kymograph analysis to quantify lamellipodia dynamics in HUVEC expressing GFP-actin. *A*. A line was drawn perpendicular to the edge of a cell expressing GFP-actin to generate a kymograph, with the x-axis representing time and the y-axis representing distance. *B*. Membrane protrusions were then identified in the kymograph and (*C*) lines were drawn from the start point to the finish point for each protrusion. *D*. Each line was then used to determine the protrusion velocity, protrusion distance, and protrusion persistence. *E*. A line from the end of the protrusion phase to the point at which the lamellipodium had completely withdrawn was also drawn, and the bounding rectangle data produced the withdrawal distance, velocity, and time.
(TIFF)

S2 Fig Kymograph analysis used to assess the lateral movement of actin stress fibers in HUVEC expressing GFP-actin. *A*. A line was drawn across the center of the cell, and a kymograph (*B*) was generated, with distance in the x-axis and time in the y-axis. *C*. Lines were superimposed over areas representing actin stress fibers, at 5 or 10-minute intervals, and the geometric data from the lines were used to calculate the number of stress fibers present at each time point and the velocity of lateral movement of stress fibers.
(PDF)

S3 Fig GFP-actin expressed in HUVEC incorporates into the actin cytoskeleton and does not affect changes in barrier function caused by thrombin or S1P. *A*. In some areas, expression efficiency was as high as 95% (scale bar = 100 μm). *B*. Western blot for β-actin (left) or GFP (right) using lysates from HUVEC expressing GFP-actin or mock-transfected cells. The top arrow shows GFP-actin and the bottom arrow shows native actin. *C*. Higher power view of GFP-actin labeling in paraformaldehyde-fixed cells. Scale bar = 20 μm. *D*. Alexafluor-594-phalloidin labeling in the same cells. *E*. Overlay of GFP-actin and Alexafluor-594-phalloidin, with nuclei labeled by Hoechst 33342 (blue). All blots and images are representative of at least 3 separate experiments. *F*. Thrombin elicited a similar change in TER in HUVEC transfected with GFP-actin plasmid and mock-transfected cells. *G*. S1P (2 μM) increased TER in both GFP- and mock-transfected HUVEC.
(TIFF)

S4 Fig Endothelial lamellipodia protrusion and withdrawal characteristics before and after treatment with thrombin. *A*. Protrusion distance. *B*. Protrusion persistence. *C*. Protrusion velocity. *D*. Withdrawal distance. *E*. Withdrawal time. *F*. Withdrawal velocity. N = 9 cells studied.
(TIFF)

S5 Fig Velocity of lateral movement of actin stress fibers in endothelial cells before and after treatment with thrombin. Thrombin did not significantly change this stress fiber lateral velocity. N = 9 cells studied.
(TIFF)

S6 Fig Endothelial lamellipodia protrusion and withdrawal characteristics before and after treatment with S1P. *A*. Protrusion distance. *B*. Protrusion velocity. *C*. Withdrawal distance. *D*. Withdrawal velocity. *E*. Number of protrusions (% of total) that had a withdrawal time lasting 5 minutes or more. *P<0.05, baseline vs. S1P. N = 9 cells studied.
(TIFF)

S7 Fig Endothelial lamellipodia protrusion and withdrawal characteristics before and after treatment with 1 U/ml thrombin, and then 2 μM S1P. *A*. Protrusion distance. *B*. Protrusion persistence. *C*. Protrusion velocity. *D*. Withdrawal distance. *E*. Withdrawal time. *F*. Withdrawal velocity. G. Number of protrusions (% of total) that had a withdrawal time lasting 5 minutes or more. *P<0.05 versus the 20 min time point (when S1P was added). †P<0.05, S1P+thrombin vs. thrombin alone. N = 9 cells studied.
(TIFF)

S8 Fig (-)Blebbistatin and lamellipodia protrusions. *A*. Protrusion distance. *B*. Protrusion persistence. *C*. Protrusion velocity. *D*. Withdrawal distance. *E*. Withdrawal time. *F*. Withdrawal velocity. *G*. Number of protrusions (% of total) that had a withdrawal time lasting 5 min or more. *P<0.05 vs. baseline (0 min time point). N = 9 cells studied.
(TIFF)

S9 Fig (+)Blebbistatin and lamellipodia protrusions. *A*. Protrusion distance. *B*. Protrusion persistence. *C*. Protrusion velocity. *D*. Withdrawal distance. *E*. Withdrawal time. *F*. Withdrawal velocity. *G*. Number of protrusions (% of total) that had a withdrawal time lasting 5 min or more. N = 9 cells studied.
(TIFF)

S10 Fig NSC23766 and lamellipodia protrusions. *A*. Protrusion distance. *B*. Protrusion persistence. *C*. Protrusion velocity. *D*. Withdrawal distance. *E*. Withdrawal time. *F*. Withdrawal velocity. *G*. Number of protrusions (% of total) that had a withdrawal time lasting 5 min or more. *H*. Lateral velocity of actin stress fibers. *I*. Number of actin stress fibers. N = 9 cells studied.
(TIFF)

S11 Fig Overexpression of wild-type (WT) or dominant-negative (DN) Rac1 and lamellipodia dynamics. Expression of GFP served as control. *A*. Protrusion persistence. *B*. Protrusion velocity. *C*. Withdrawal distance. *D*. Withdrawal velocity. *P<0.05 between the indicated groups. N = 9 cells studied for each group.
(TIFF)

S1 Movie Dynamic motion of GFP-actin in endothelial cells during baseline conditions. Local lamellipodia that form at the edges of cells, and actin clouds that form in the cytoplasm are shown. Actin fibers are apparent mainly in the cell periphery but can also be found in the central area of some cells. Elapsed time is shown in the bottom right corner.
(AVI)

S2 Movie Impact of 1 U/ml thrombin treatment on dynamics of the GFP-actin cytoskeleton in endothelial cells. This movie is a continuation of the same cells shown in S1 Movie, which were treated with thrombin at the 60 min time point. Shortly after thrombin treatment, actin appears at the cell edges and on vesicles. Soon after, stress fibers assemble in the cytoplasm and transverse arc fibers moved inward. A gap at the junction of three cells also opens and closes. Elapsed time is shown in the bottom right corner. Due to fading of the GFP intensity over the time course of S1 Movie, the brightness and contrast were readjusted to optimize view of the GFP-actin structures.
(AVI)

S3 Movie Gap formation and closure after thrombin treatment of endothelial cells expressing GFP-actin. A cortical actin fiber that existed prior to the addition of thrombin is shown by the two arrows at the beginning of the movie and later just prior to the opening of the gap. Note that just after the gap starts to form, this and fibers that terminate at the edge of the cell retract centripetally. Local lamellipodia close the gap, and after closure, additional small local lamellipodia continue to protrude and withdraw in the closed space.
(AVI)

S4 Movie S1P-induced endothelial cell lamellipodia formation. In endothelial cells expressing GFP-actin, within 1 minute after the addition of 2 μM S1P, lamellipodia began to protrude in a coordinated fashion along most of the edges of adjacent cells. This activity was followed by some cell retraction and protrusion that was less coordinated and differed from cell to cell.
(AVI)

S5 Movie Impact of thrombin, followed by S1P 20 min later, on GFP-actin structures in endothelial cells. Local lamellipodia and actin fibers are visible in the cells expressing GFP-actin. The large areas with no signal were occupied by cells not expressing the vector. At 20 min, 1 U/ml thrombin was added, and a reduction in lamellipodia protrusions is apparent, followed by retraction of some cells. At 40 min, 2 μM S1P was added, followed by a rapid burst of local lamellipodia on most cells.
(AVI)

S6 Movie Dynamics of VE-cadherin-GFP in endothelial cells during baseline conditions. VE-cadherin was concentrated mainly at the periphery (presumably cell-cell junctions) and in cytoplasmic vesicles. Local lamellipodia were detected protruding beyond the VE-cadherin belt at the cell periphery. VE-cadherin-GFP was not typically abundant in the lamellipodia, although in some cases there appeared to be VE-cadherin-GFP in structures resembling filopodia that formed during local lamellipodia withdrawal.
(AVI)

S7 Movie Local lamellipodia are apparent in HUVEC expressing VE-cadherin-GFP. Thrombin (1 U/ml) inhibits local lamellipodia protrusions, followed by disruption of the peripheral VE-cadherin-GFP belt.
(AVI)

S8 Movie S1P (2 μM) had little impact on VE-cadherin-GFP localization, however did cause protrusions of the cell membrane well beyond the VE-cadherin belt at intercellular junctions. (AVI)

S9 Movie Inhibition of myosin II with 100 μM (-)Blebbistatin inhibits local lamellipodia in HUVEC expressing GFP-actin. For comparison, the right panel shows HUVEC treated with 100 μM (+)Blebbistatin over the same time period. Blebbistatin was added at the 30 min time point.
(AVI)

S10 Movie Impact of the Rac1 inhibitor NSC23766 on the actin cytoskeleton in HUVEC expressing GFP-actin. NSC23766 (50 μM) was added at the 10 min time point. (AVI)

S11 Movie Visualization of cell protrusions at cell edges using HUVEC overexpressing GFP, GFP-Rac1-WT, or GFP-Rac1-DN. For cells expressing each vector, 10-min time-lapse image sets as the ones shown were used to determine lamellipodia protrusion dynamics.
(AVI)
