# Supplementary figures and images for: Involvement of Local Lamellipodia in Endothelial Barrier Function
Source: PLoS One. 2015 Feb 6;10(2):e0117970. doi: 10.1371/journal.pone.0117970 (PMC4320108; doi:10.1371/journal.pone.0117970)

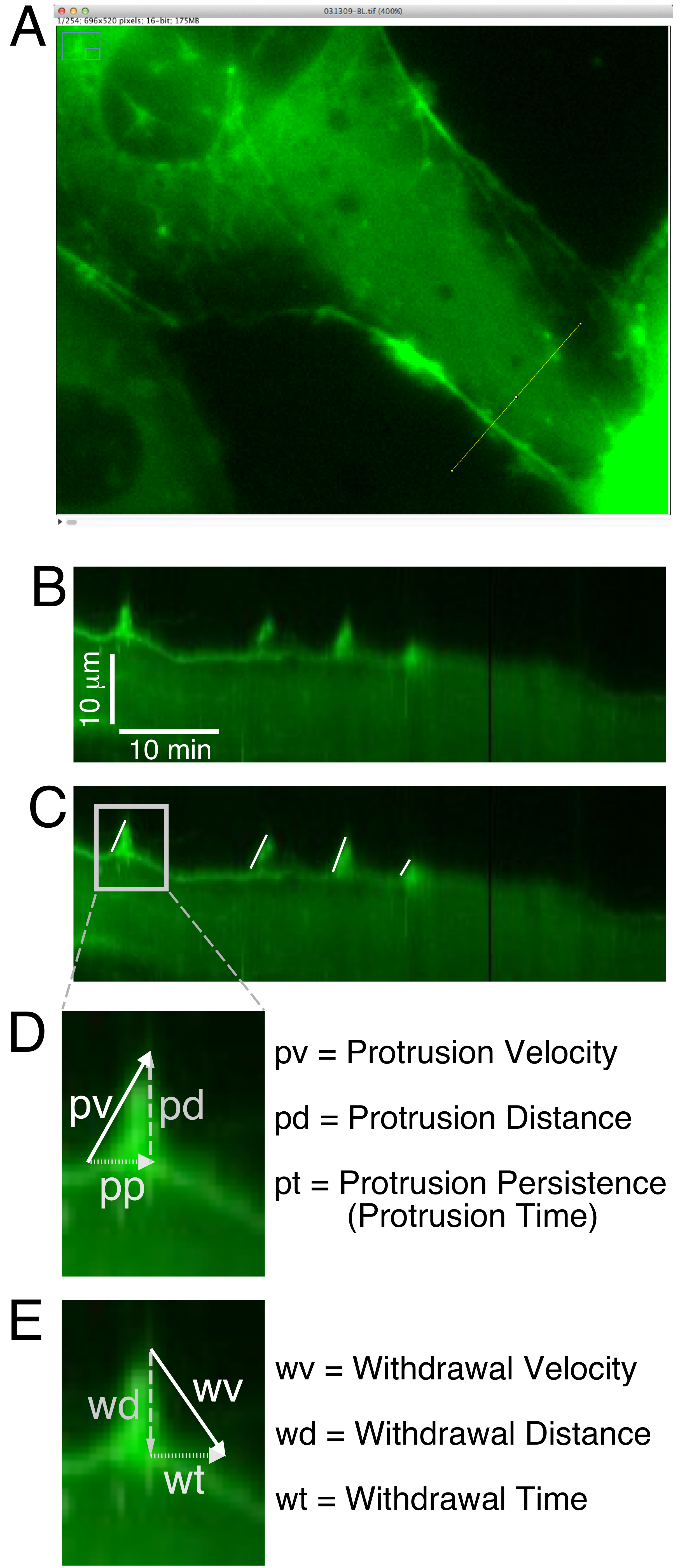

Supplement: S1 Fig — A. A line was drawn perpendicular to the edge of a cell expressing GFP-actin to generate a kymograph, with the x-axis representing time and the y-axis representing distance. B. Membrane protrusions were then identified in the kymograph and (C) lines were drawn from the start point to the finish point for each protrusion. D. Each line was then used to determine the protrusion velocity, protrusion distance, and protrusion persistence. E. A line from the end of the protrusion phase to the point at which the lamellipodium had completely withdrawn was also drawn, and the bounding rectangle data produced the withdrawal distance, velocity, and time. (TIFF) [file pone.0117970.s001.tiff]

**A**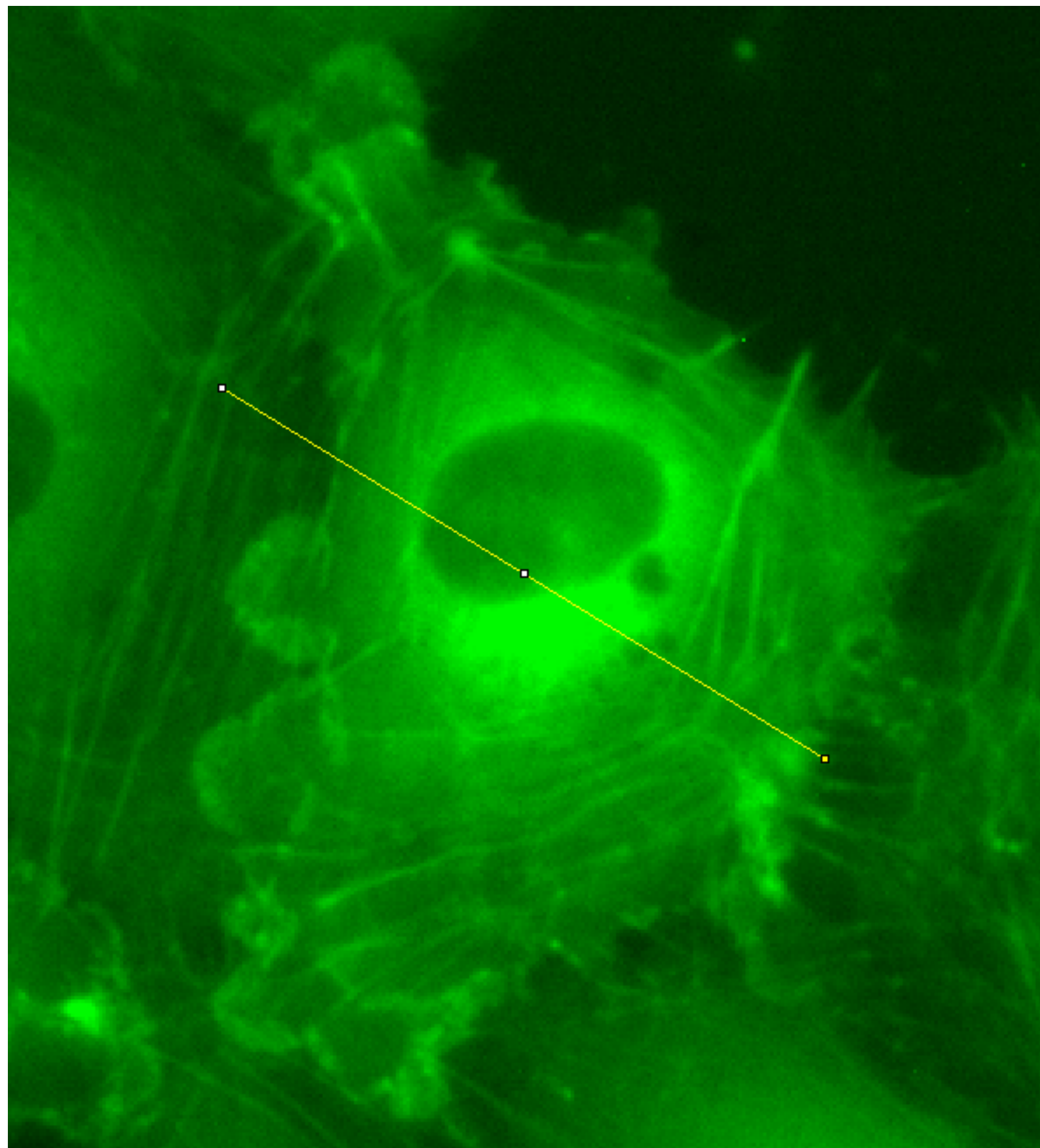**B**

20  $\mu\text{m}$   
20 min.

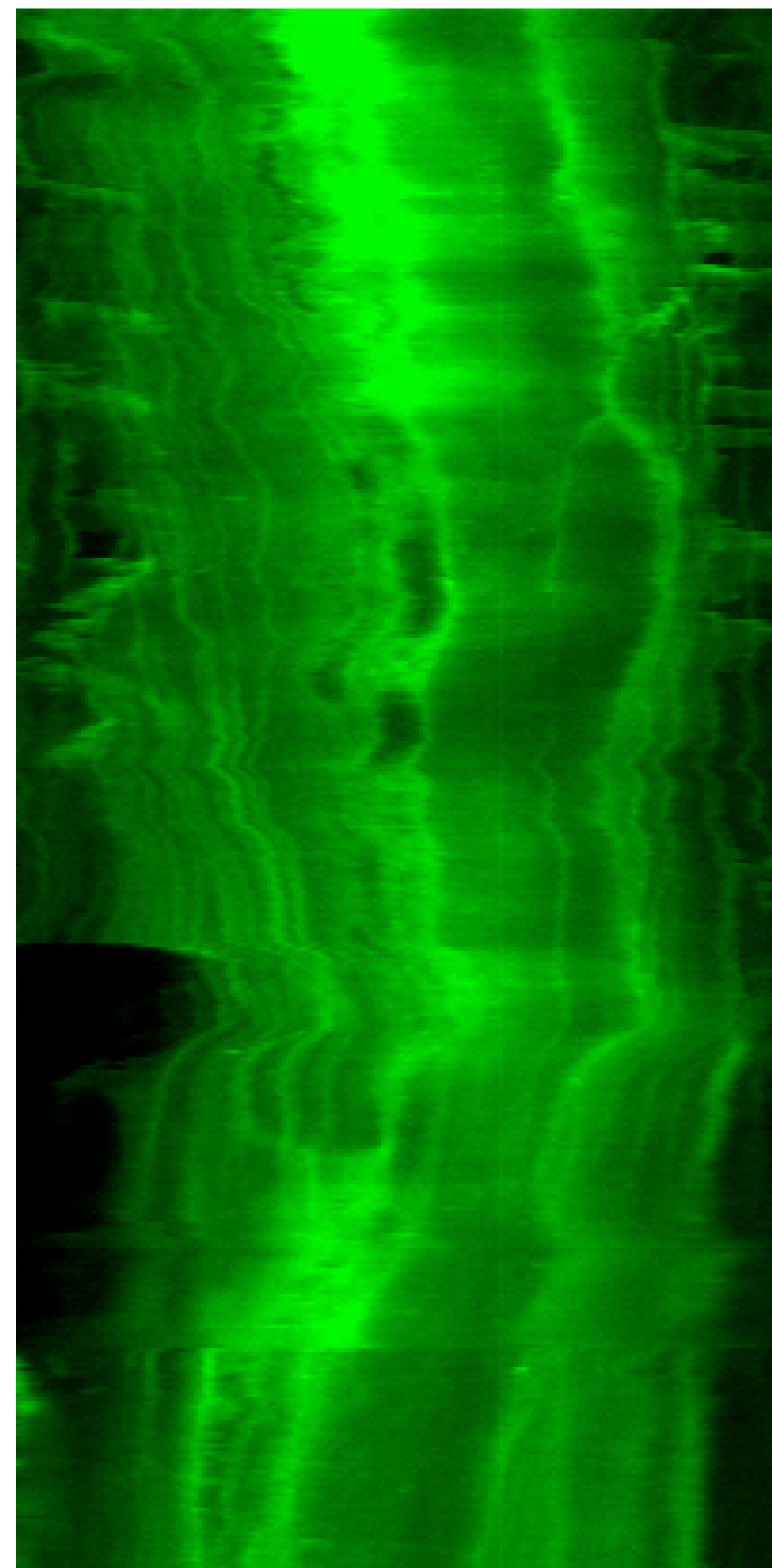**C**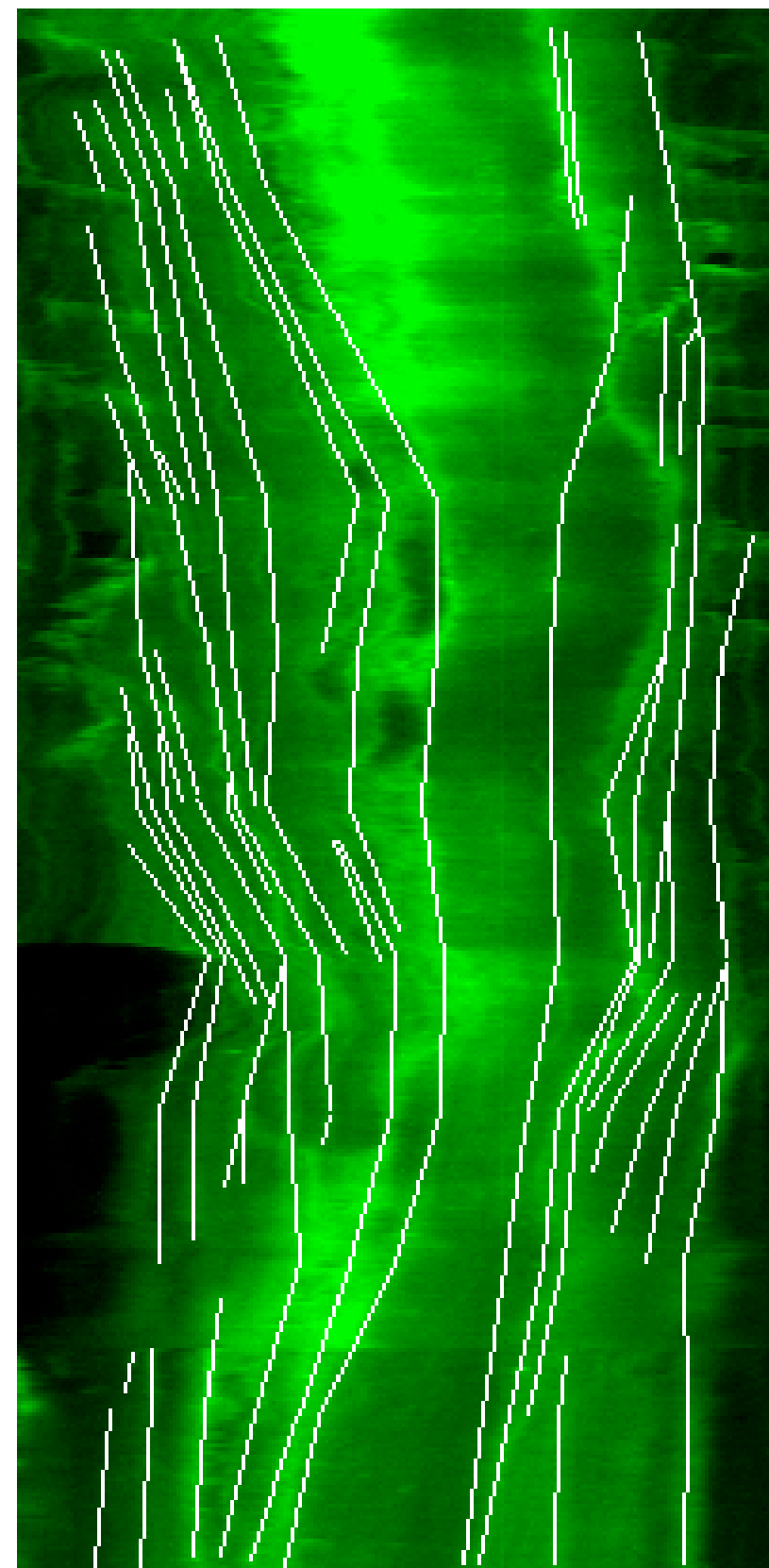

Supplement: S2 Fig — A. A line was drawn across the center of the cell, and a kymograph (B) was generated, with distance in the x-axis and time in the y-axis. C. Lines were superimposed over areas representing actin stress fibers, at 5 or 10-minute intervals, and the geometric data from the lines were used to calculate the number of stress fibers present at each time point and the velocity of lateral movement of stress fibers. (PDF) [file pone.0117970.s002.pdf]

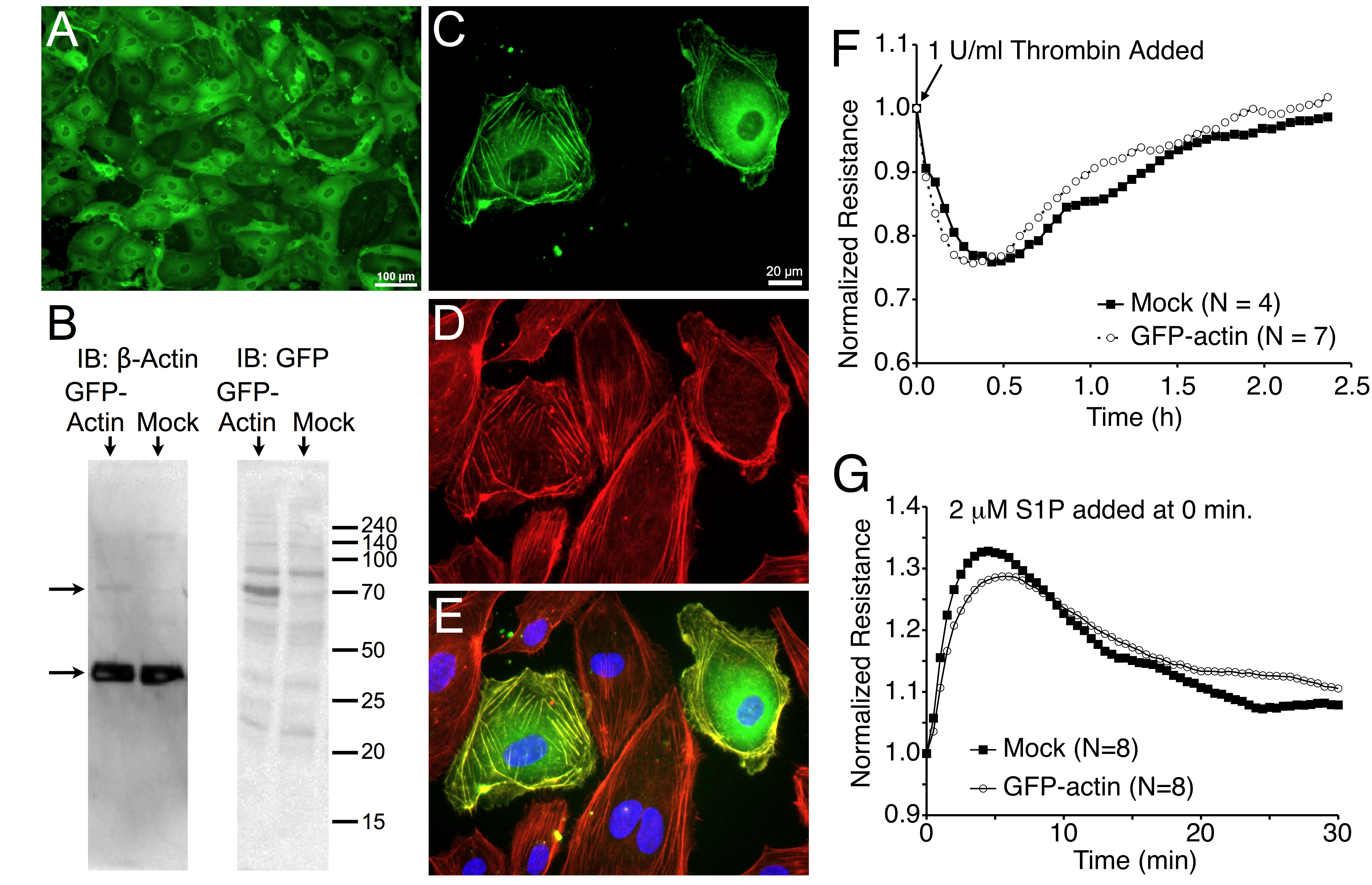

Supplement: S3 Fig — A. In some areas, expression efficiency was as high as 95% (scale bar = 100 μm). B. Western blot for β-actin (left) or GFP (right) using lysates from HUVEC expressing GFP-actin or mock-transfected cells. The top arrow shows GFP-actin and the bottom arrow shows native actin. C. Higher power view of GFP-actin labeling in paraformaldehyde-fixed cells. Scale bar = 20 μm. D. Alexafluor-594-phalloidin labeling in the same cells. E. Overlay of GFP-actin and Alexafluor-594-phalloidin, with nuclei labeled by Hoechst 33342 (blue). All blots and images are representative of at least 3 separate experiments. F. Thrombin elicited a similar change in TER in HUVEC transfected with GFP-actin plasmid and mock-transfected cells. G. S1P (2 μM) increased TER in both GFP- and mock-transfected HUVEC. (TIFF) [file pone.0117970.s003.tiff]

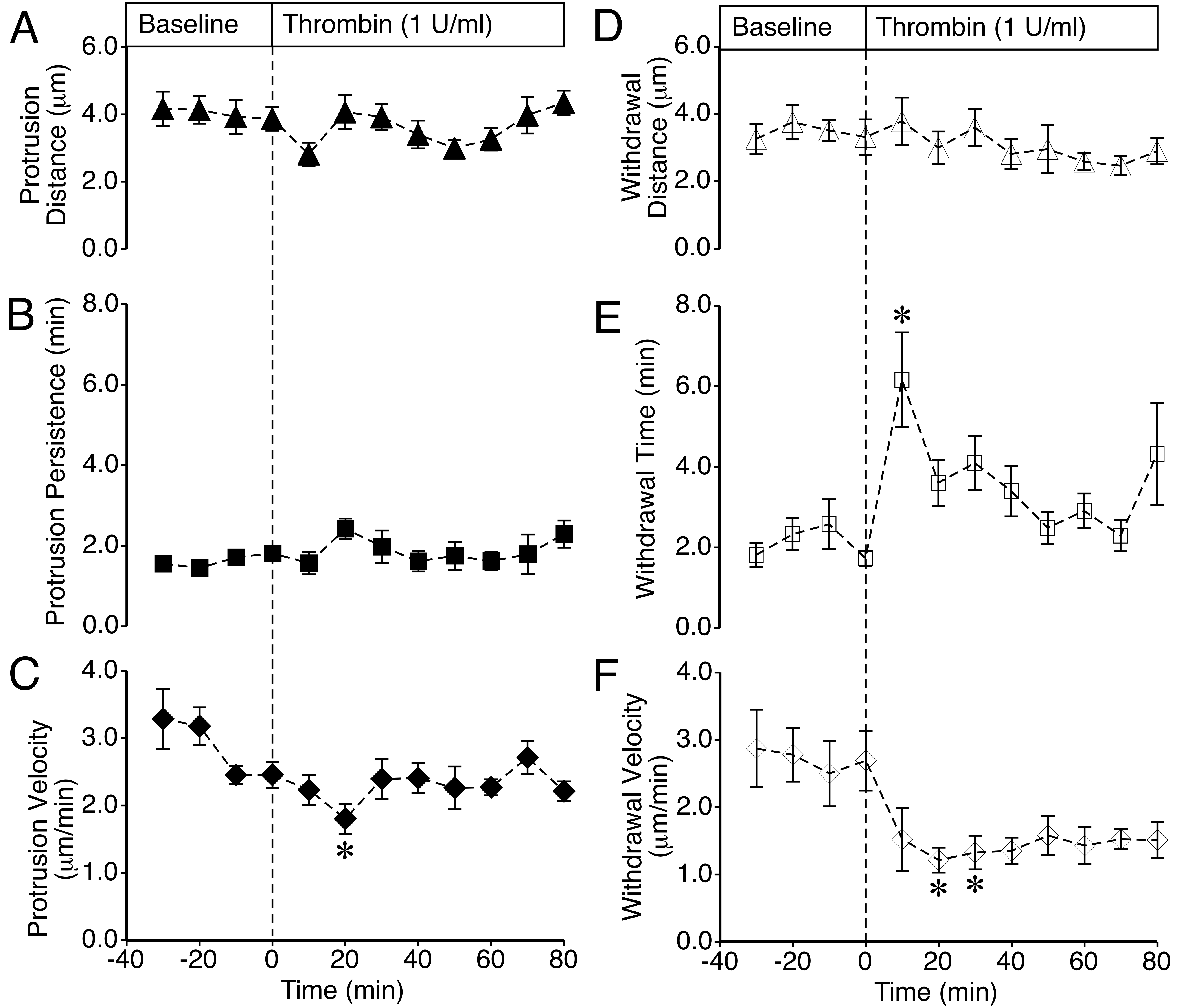

Supplement: S4 Fig — A. Protrusion distance. B. Protrusion persistence. C. Protrusion velocity. D. Withdrawal distance. E. Withdrawal time. F. Withdrawal velocity. N = 9 cells studied. (TIFF) [file pone.0117970.s004.tiff]

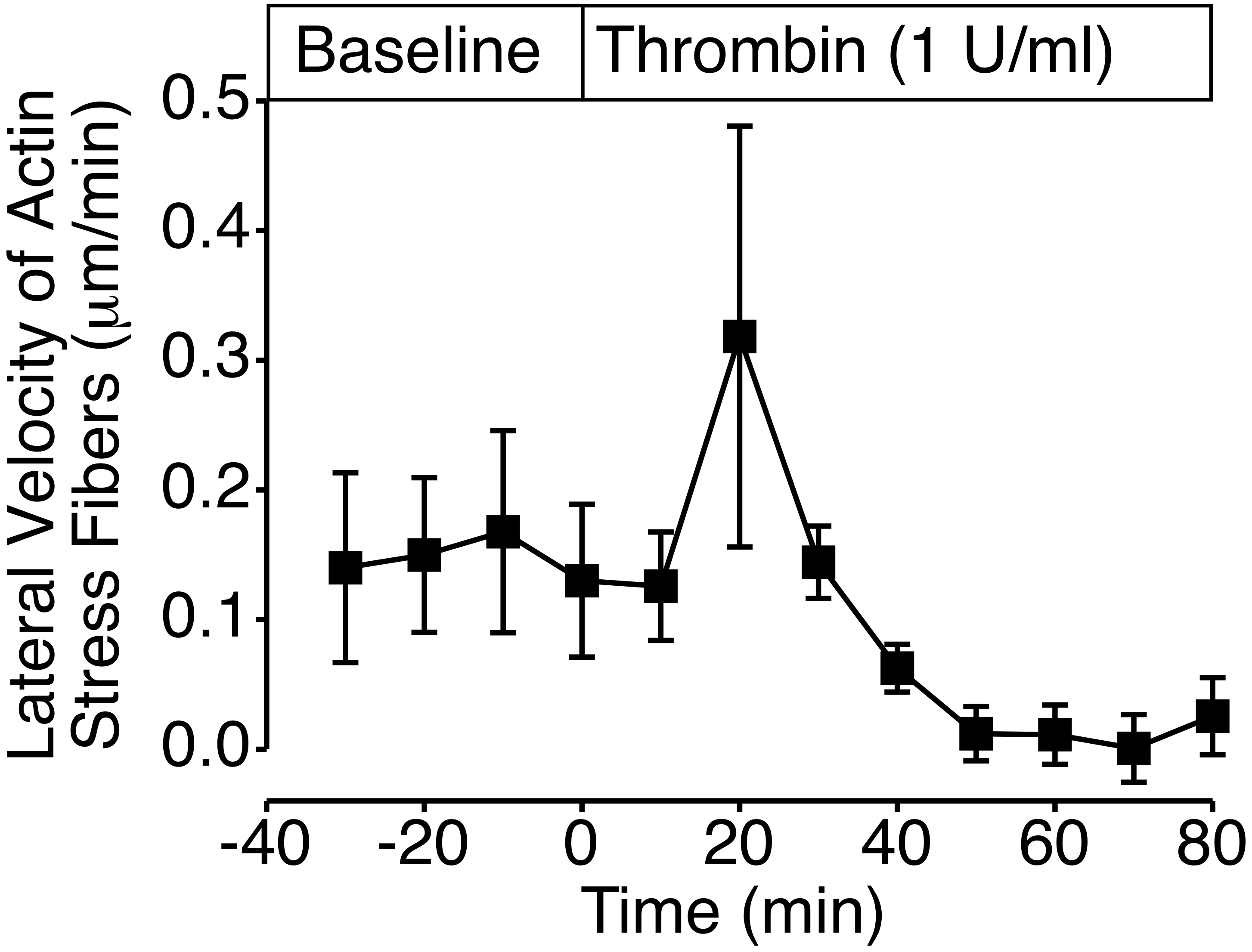

Supplement: S5 Fig — Thrombin did not significantly change this stress fiber lateral velocity. N = 9 cells studied. (TIFF) [file pone.0117970.s005.tiff]

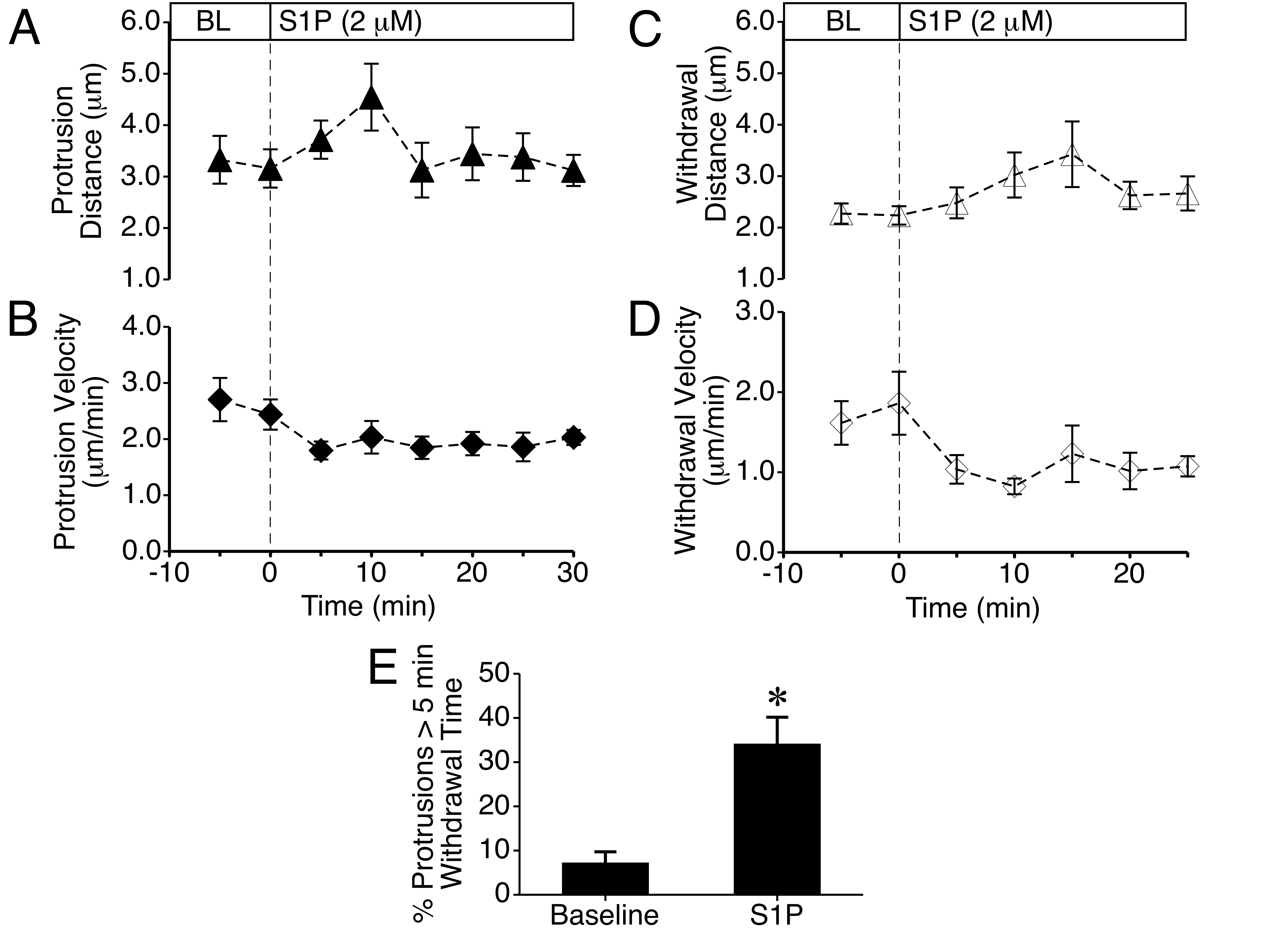

Supplement: S6 Fig — A. Protrusion distance. B. Protrusion velocity. C. Withdrawal distance. D. Withdrawal velocity. E. Number of protrusions (% of total) that had a withdrawal time lasting 5 minutes or more. *P<0.05, baseline vs. S1P. N = 9 cells studied. (TIFF) [file pone.0117970.s006.tiff]

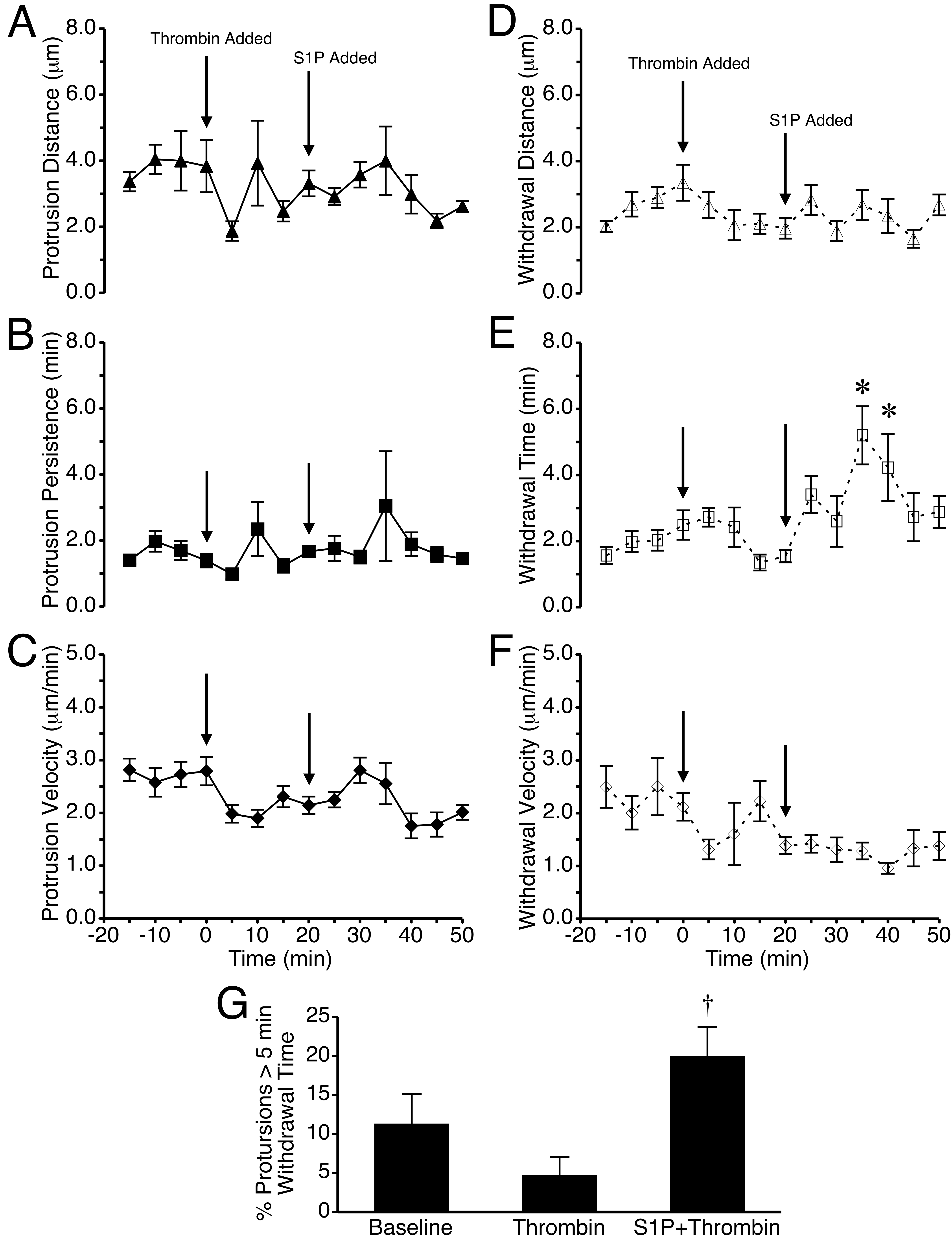

Supplement: S7 Fig — A. Protrusion distance. B. Protrusion persistence. C. Protrusion velocity. D. Withdrawal distance. E. Withdrawal time. F. Withdrawal velocity. G. Number of protrusions (% of total) that had a withdrawal time lasting 5 minutes or more. *P<0.05 versus the 20 min time point (when S1P was added). †P<0.05, S1P+thrombin vs. thrombin alone. N = 9 cells studied. (TIFF) [file pone.0117970.s007.tiff]

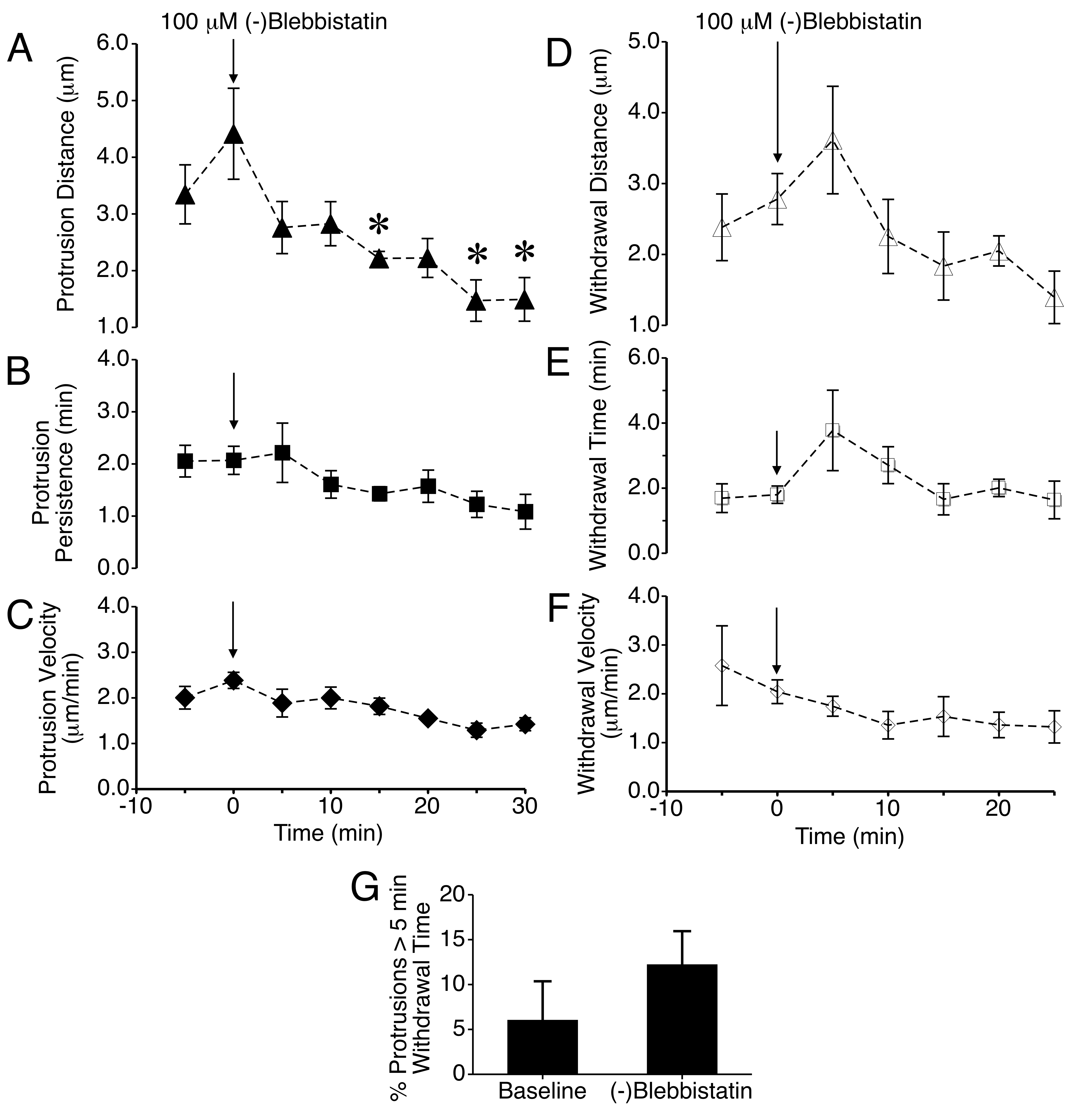

Supplement: S8 Fig — A. Protrusion distance. B. Protrusion persistence. C. Protrusion velocity. D. Withdrawal distance. E. Withdrawal time. F. Withdrawal velocity. G. Number of protrusions (% of total) that had a withdrawal time lasting 5 min or more. *P<0.05 vs. baseline (0 min time point). N = 9 cells studied. (TIFF) [file pone.0117970.s008.tiff]

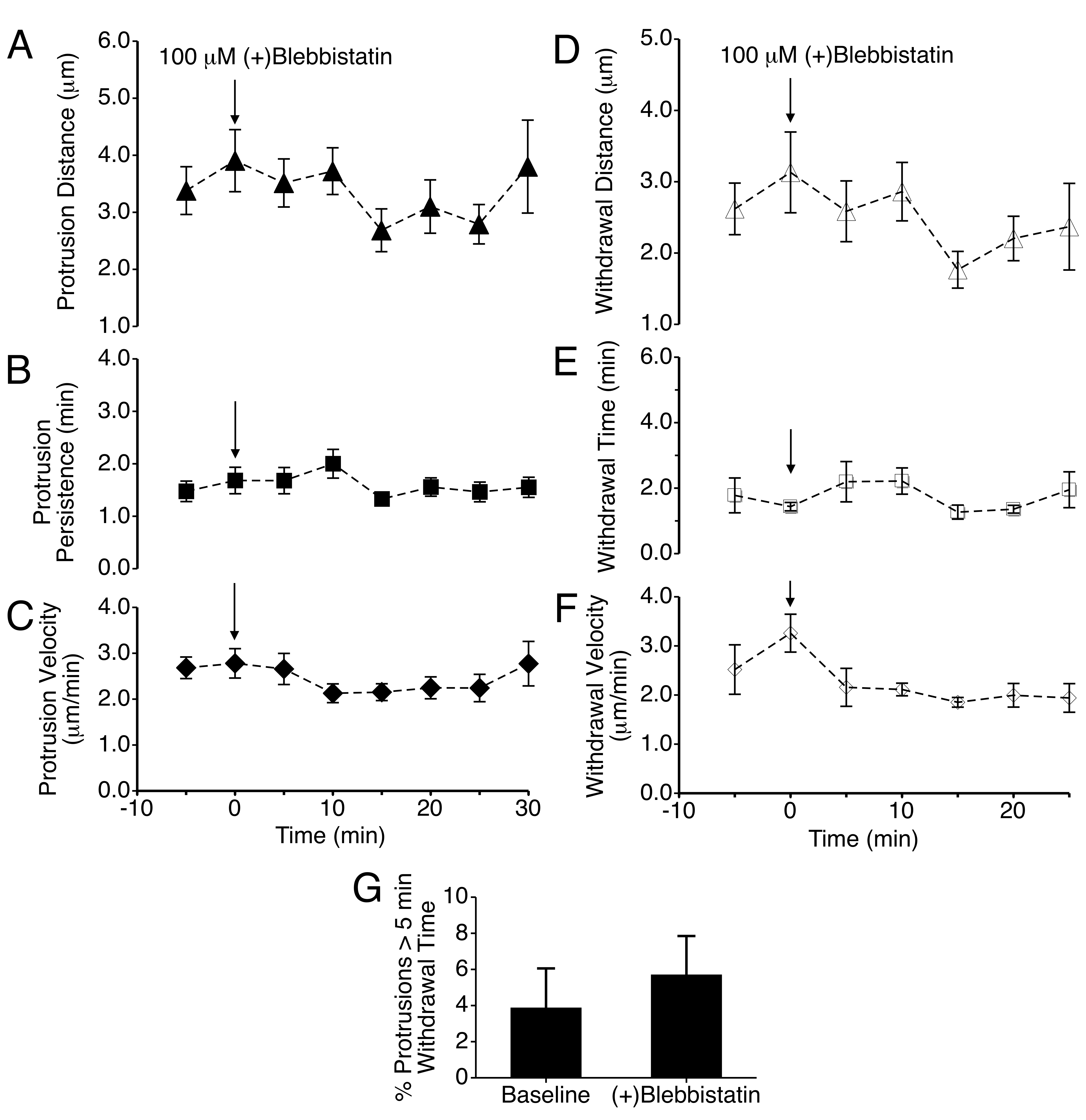

Supplement: S9 Fig — A. Protrusion distance. B. Protrusion persistence. C. Protrusion velocity. D. Withdrawal distance. E. Withdrawal time. F. Withdrawal velocity. G. Number of protrusions (% of total) that had a withdrawal time lasting 5 min or more. N = 9 cells studied. (TIFF) [file pone.0117970.s009.tiff]

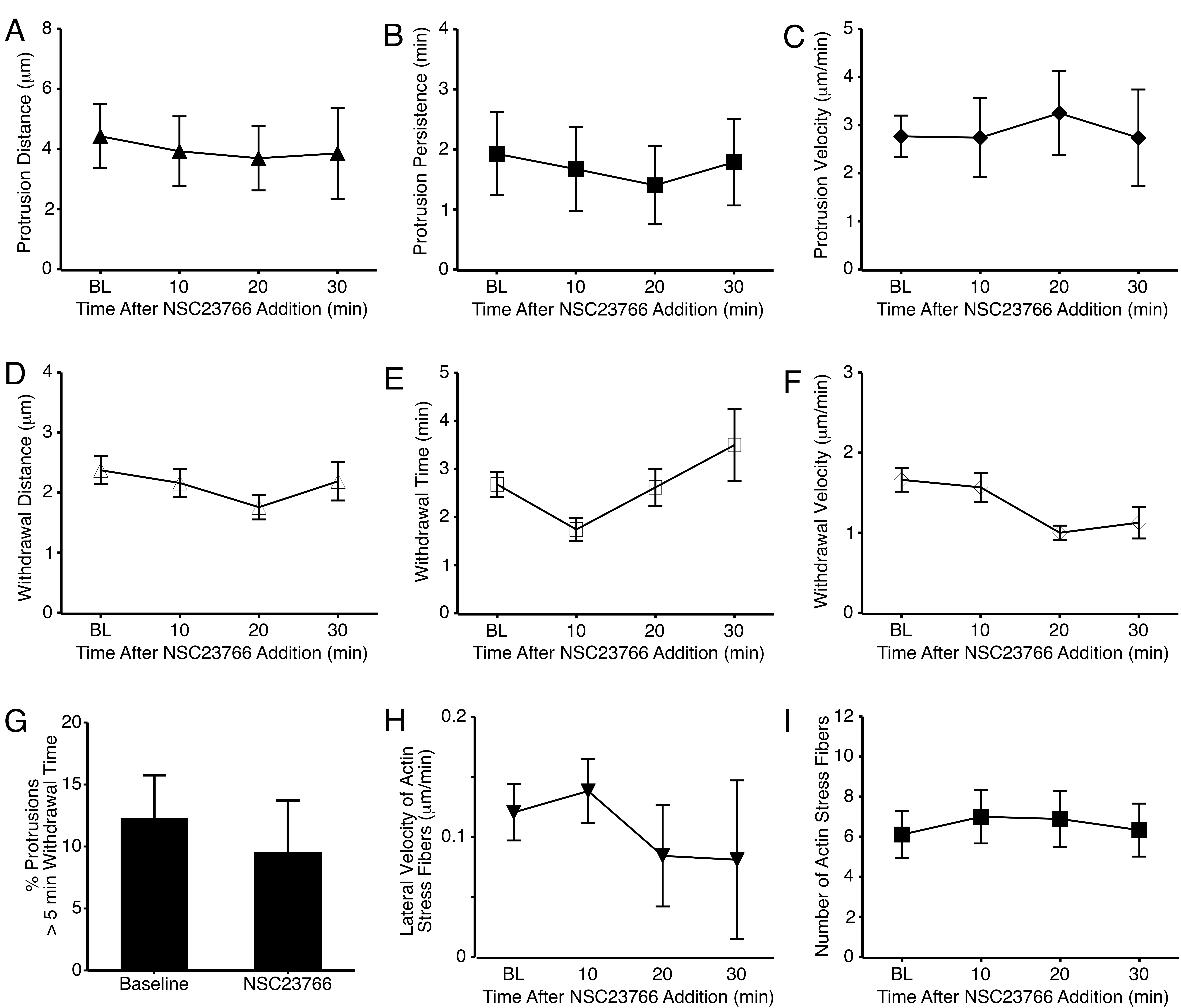

Supplement: S10 Fig — A. Protrusion distance. B. Protrusion persistence. C. Protrusion velocity. D. Withdrawal distance. E. Withdrawal time. F. Withdrawal velocity. G. Number of protrusions (% of total) that had a withdrawal time lasting 5 min or more. H. Lateral velocity of actin stress fibers. I. Number of actin stress fibers. N = 9 cells studied. (TIFF) [file pone.0117970.s010.tiff]

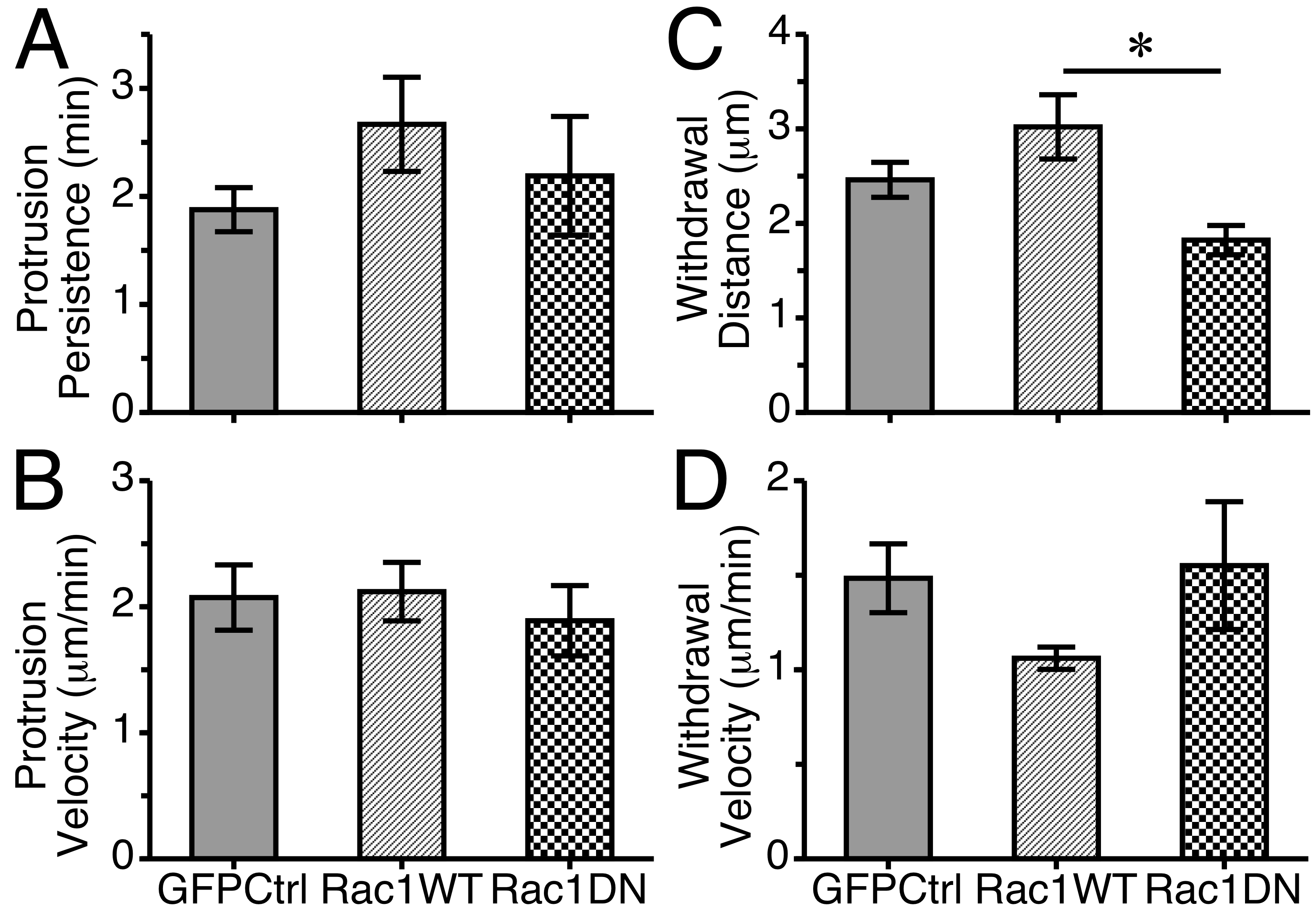

Supplement: S11 Fig — Expression of GFP served as control. A. Protrusion persistence. B. Protrusion velocity. C. Withdrawal distance. D. Withdrawal velocity. *P<0.05 between the indicated groups. N = 9 cells studied for each group. (TIFF) [file pone.0117970.s011.tiff]
